# NF-κB/Lipocalin 2 Signaling Pathway Mitigates the Chemoresistance of BRAFV600E-Mutant Colorectal Cancer to Cisplatin by Promoting Ferroptosis

**DOI:** 10.3390/cancers18162552

**Published:** 2026-08-09

**Authors:** Meibao Feng, Xuesong Wu, Li Jiang, Jinyan Huang, Jing Zhang, Pei Chen, Chengdong Chang

**Affiliations:** 1 Department of Pathology, School of Medicine, The First Affiliated Hospital of Zhejiang University, Hangzhou 310003, China; 2 Laboratory of Animal Research Center, School of Medicine, The First Affiliated Hospital of Zhejiang University, Hangzhou 310003, China; 3 Biomedical Big Data Center, School of Medicine, The First Affiliated Hospital of Zhejiang University, Hangzhou 310003, China

**Keywords:** BRAFV600E, lipocalin 2, chemoresistance, ferroptosis, apoptosis, colorectal cancer

## Abstract

**Background**: Colorectal cancers (CRCs) harboring the BRAFV600E (V600E) mutation exhibit aggressive clinical behavior and chemotherapy resistance, yet the underlying mechanisms remain poorly understood. Ferroptosis, which is driven by iron-dependent lipid peroxidation, has emerged as a potential therapeutic vulnerability. This study aimed to explore whether the NF-κB/Lipocalin 2 (LCN2) pathway modulates cisplatin sensitivity through Fenton-reaction-induced ferroptosis in BRAFV600E-overexpressing CRC cells. **Methods**: BRAF mutation status and the expression of LCN2, PTGS2, and cleaved caspase3 were examined in clinical CRC specimens by immunohistochemistry. The correlations between ferroptosis and apoptosis markers and 5-year survival rate were evaluated in TCGA datasets. CRC cells with LCN2 knockdown/knockout or BRAF/V600E/LCN2 overexpression were established to assess the proliferation, lipid metabolism, iron levels, and NF-κB/LCN2 signaling under cisplatin treatment. In vivo studies were employed with *BALB/c* xenograft models. **Results**: V600E-mutant clinical specimens exhibit significantly reduced expression of the ferroptosis marker PTGS2, and the iron metabolism regulators LCN2. Within a KRAS-mutant cellular model, V600E overexpression attenuated cisplatin-induced ferroptosis through suppression of the NF-κB/LCN2 signaling axis, leading to impairment of Fenton-reaction-mediated lipid peroxidation. Restoration of LCN2 expression re-sensitized V600E-overexpressing cells to cisplatin both in vitro and in vivo. Interestingly, inhibition of apoptosis contributes to the resistance of cisplatin induced ferroptosis in V600E overexpression cells, implying a crosstalk between ferroptosis and apoptosis within the therapeutic resistance. **Conclusions**: Our findings show that the NF-κB/LCN2 axis drives Fenton-reaction-induced ferroptosis to promote the vulnerability of V600E overexpression CRC cells within a KRAS-mutant background to cisplatin. LCN2 restoration partially overcomes V600E overexpression resistance both in vitro and in vivo, suggesting LCN2 as a promising therapeutic target. The crosstalk between ferroptosis and apoptosis may offer potential strategies to overcome chemotherapy resistance of this high-risk CRC subtype.

## Simple Summary

Colorectal cancer (CRC) driven by the BRAFV600E (V600E) mutation represents one of the most clinically challenging subtypes of the disease, characterized by rapid progression and a striking resistance to conventional chemotherapy. Despite its poor prognosis, the molecular mechanisms have remained unclarified. In this study, we found a key role for NF-κB/Lipocalin 2 (LCN2)-signaling-pathway-induced ferroptosis in modulating cisplatin sensitivity of V600E-overexpressing CRC cells to cisplatin within a KRAS-mutant cellular background. The results show that V600E overexpression cells exhibit suppressed NF-κB/LCN2 activation and insensitivity to cisplatin treatment. The NF-κB/LCN2 signaling could induce ferroptosis through increasing the accumulation of iron, leading to activation of the Fenton reaction and increasing ROS levels. Restoring LCN2 expression in V600E overexpression cells could partially attenuate the inhibition of ferroptosis and chemoresistance of cisplatin. Interestingly, we further found an unexpected interplay between ferroptosis and apoptosis, which is also regulated by LCN2. Taken together, these findings imply LCN2 as a potential therapeutic target. Aiming the crosstalk between ferroptosis and apoptosis may offer a potential strategy to overcome chemotherapy resistance of this high-risk CRC subtype.

## 1. Introduction

Colorectal cancer (CRC) represents a major global health burden and a leading cause of global cancer mortality. While incidence and mortality rates for gastric, liver, and esophageal cancers have declined, those for CRC continue to rise significantly [1]. Despite advances in systemic therapies, elucidating the mechanisms of tumorigenesis and identifying novel therapeutic targets remain urgent priorities. The BRAFV600E mutation is observed in approximately 8–10% of CRC [2] and is associated with aggressive clinicopathological features, including microsatellite instability, poor differentiation, and increased lymph node metastasis [3,4]. Consequently, patients harboring this mutation exhibit markedly worse disease-free and overall survival, even following neoadjuvant chemotherapy [5,6,7,8], highlighting the significance of exploring the underlying mechanisms.

Ferroptosis, a form of regulated cell death characterized by the lethal accumulation of lipid peroxides and iron-dependent reactive oxygen species (ROS) [9,10,11], is implicated in drug resistance in solid tumors. The RAS/RAF/MEK signaling pathway is a key regulator of ferroptosis. BRAF knockout leads to resistance to erastin-induced ferroptosis [12]. Furthermore, the MEK/ERK pathway modulates NF-ĸB activation, which significantly upregulates Lipocalin 2 (LCN2) expression. Ferroptosis could propagate between cells in a wave-like manner, indicating its potent cytotoxic potential against adjacent cells [13,14] Since its discovery, ferroptosis has emerged as a promising target for anti-tumor therapy, particularly for overcoming chemotherapy resistance [15,16]. However, the role BRAFV600E plays in ferroptosis remains poorly clarified.

Lipocalin 2 (LCN2) is a multifaceted protein that regulates iron homeostasis and inflammatory responses. It functions as a key component of innate immunity by sequestering iron and is a sensitive biomarker for inflammatory conditions such as inflammatory bowel disease (IBD), a known risk factor for CRC [17]. Serum LCN2 demonstrates high diagnostic accuracy for IBD, with sensitivity and specificity rates of 95% and 83%, respectively, surpassing traditional markers like ESR and CRP. It can be detected in urine, blood, and excretions, serving as a biomarker for human inflammatory diseases [18,19] Research indicates that LCN2 could enhance chemosensitivity and inhibit epithelial–mesenchymal transition (EMT), thereby reducing proliferation, tumorigenicity, and metastasis [20,21]. Furthermore, LCN2 has been shown to be associated with improved tumor differentiation and higher five-year survival. Notably, ferroptosis, as observed in processes like EMT, has been established as a potent mechanism for chemoresistance in cancer [22,23,24].

Moreover, drug-tolerant persister cells (PCCs) play a pivotal role in multidrug resistance (MDR) development due to the close association between PCC and EMT and the limited number of PCCs. Moreover, as PCCs exhibit high drug resistance, conventional cytotoxic drugs show poor effect. Ferroptosis, however, demonstrates remarkable potential to eliminate EMT cells by modulating intracellular redox homeostasis, offering a novel strategy to overcome cancer MDR [25,26,27]. The relationship between LCN2 and ROS appears complicated. Researches indicate that LCN2 could promote tumor growth through inducing ROS production and enhancing DNA damage in adjacent normal cells [28]. Conversely, LCN2 knockout has been reported to ameliorate erastin-mediated ferroptosis and increases cisplatin vulnerability in breast cancer cells [29]. Moreover, LCN2 is closely associated with apoptosis [30], suggesting its role in ROS modulation requires further investigation.

Published results demonstrate that LCN2 can inhibit CRC metastasis and invasion by attenuating NF-ĸB activity, subsequently suppressing EMT progression [20]. Based on the previously established transcriptional regulation of LCN2 by NF-ĸB [31], and the established functions of LCN2 in iron homeostasis, this study aims to explore whether NF-ĸB/LCN2 signaling contributes to cisplatin resistance through dysregulated iron homeostasis and ferroptosis in BRAFV600E mutant CRC.

## 2. Materials and Methods

### 2.1. Cell Culture and Reagents

The human CRC cell lines of HT-29, SW620, DLD1 and 293FT cells were generous gifts from Professor Maode Lai (department of pathology, school of medicine, Zhejiang University). They were respectively cultured in RPMI 1640 and Dulbecco’s Modified Eagle’s Medium (DMEM) (Gibco, Grand Island, NY, USA) supplemented with 10% fetal bovine serum (FBS, Excell Bioscience, Shanghai, China) and mycoplasma removal agent (1:100) and grown at 37 °C under 5% CO_2_. HT29 cells with LCN2 knockdown or knockout (designated as HT29-shLCN2 and HT29-LCN2-KO; vector control-SHB), DLD1 cells with expression of BRAF and BRAFV600E (designated as over-BRAF/V600E; vector control-OB) and SW620 cells that stably expressed LCN2 (designated as OVER-LCN2; vector control-OB) were established by lentiviral shuttle vector. Cisplatin (cis, Cat. No. HY-17394) was purchased from Med Chem Express (MCE, Hao yuan Bioscience, Shanghai, China), and cisplatin was incubated with the corresponding cells at a concentration of 30 μM for 24–48 h. H2DCFDA (Cat. No. HY-D0940) and ZVAD-FMK (Cat. No. HY-16658B) were from Med Chem Express (MCE, Hao yuan Bioscience, Shanghai, China). Tetramethylrhodamine (TMRM, I34361) was obtained from Invitrogen (Thermo Fisher Scientific, Waltham, MA, USA).

### 2.2. Plasmid Construction and Viral Transduction

For gene knockdown, shRNA sequences targeting human BRAF or LCN2 were cloned into the pLKO.1-puro lentiviral vector. For overexpression, the full-length coding sequences of human LCN2, wild-type BRAF or BRAFV600E were cloned into the pLVX-puro vector. The lentiviral shuttle vector LV242-LCN2, BRAF and BRAFV600E was constructed to stably overexpress LCN2 or BRAF. BRAFV600E plasmids were generated by LeQi-Biomedicine (Hangzhou, China) Co. Ltd. Lentiviruses were produced in 293T cells using the psPAX2 and pMD2.G packaging plasmids. Target cells were infected with lentivirus in the presence of 8 µg/mL polybrene and selected with 2 µg/mL puromycin for 7 days. The lentiviral shuttle vector pLKO.1 was used to express shRNA. The target sequences of shRNA were:

shLCN2-1: 5′-GAGTTCACGCTGGGCAACATTAAGA-3′;

shLCN2-2: 5′-GCTGGGCAACATTAAGAGTTA-3′.

### 2.3. Generation of the LCN2-KO Cell Line

The CRISPR/Cas9 technology was chosen to generate Lcn2-knockout (KO) HT29 cells. The LCN2 CRISPR/Cas9 KO Plasmid (h) (sc-401838), LCN2 HDR Plasmid (h) (sc-401838-HDR) and the CRISPR/Cas9 scramble plasmid (ctr, sc-418922) were obtained from Santa Cruz Biotechnology (Dallas, TX, USA). Each LCN2 CRISPR/Cas9 KO plasmid consists of a pool of 3 plasmids, each encoding the Cas9 nuclease and a target-specific 20 nt guide RNA designed for maximum knockout efficiency. Briefly, HT29 cells were co-transfected with CRISPR/Cas9 KO plasmids or the Ctr CRISPR/Cas9 plasmids and the HDR plasmids using Lipofectin 3000 (Thermo Fisher Scientific) according to the manufacturer’s instruction. The single-clonal populations were then selected by flow cytometry and puromycin over 21 days. The absence of LCN2 in the clones generated was confirmed by Western blot analysis of protein extracts.

### 2.4. Western Blot Assay

Cells were scraped, washed 3 times with PBS, and then lysed in RIPA buffer (Cat. No. P0013B), which was purchased from Beyotime Biotechnology, Shanghai, China. Subsequently, the protein concentrations were determined by the Bradford method, and aliquots of the protein samples were stored at −80 °C. Aliquots of protein extracts (15–50 μg) were separated on 10–12% SDS-polyacrylamide gels according to the protein molecular weights. Then, the proteins were electrophoretically transferred onto NC membranes (Merck Millipore, Darmstadt, Germany). After blocking with TBS-Tween 20 (TBST) containing 5% low-fat milk for 2 h at room temperature, the membranes were incubated with primary antibody overnight at 4 °C. The membranes were then incubated with secondary antibodies (Odyssey, Li-COR, Bioscience, Lincoln, NE, USA) for 1 h at room temperature. Finally, the membranes were developed with a chemiluminescence imager (Bio-Rad ChemiDoc MP system, Hercules, CA, USA).

Antibodies against BRAF (Cat No. #14814, 1:1000) were from Cell Signaling Technology (Danvers, MA, USA), PTGS2 (AF7003, 1:1000) was purchased from Affinity Biosciences (Shanghai, China), GAPDH (Cat No. 60004-1-Ig, 1:15,000) and NF-κBp65 (Cat No. 80979-1-RR, 1:20,000) were obtained from ProteinTech, Wuhan Sanying (Wuhan East Lake Hi-tech Development Zone, Wuhan, China). Ferroportin (FPN, 1:1000, NBP1-21502) antibody was purchased from Novus Biologicals (Centennial, CO, USA). Antibodies of LCN2 (ab41105, 1:1000), NF-κBp65 (phospho S536, ab76302, 1:500) and ACSL4 (ab155282, 1:1000) were from Abcam (Cambridge, UK).

### 2.5. Ferrous Iron (Fe^2+^) Level Detection and Mitochondrial Membrane Potential (MMP) Measurement

To detect intracellular Fe^2+^ and MMP levels, FerroOrange (Dojindo, Kumamoto, Japan) and TMRM (Invitrogen, Thermo Fisher Scientific, Carlsbad, CA, USA) were used according to the manufacturers’ protocols. Corresponding cells were seeded in 96-well plates and treated with cisplatin for the indicated time and stained with a final concentration of 1 μM FerroOrange and 0.1 μM TMRM, respectively, for 30 min at 37 °C protected from light, then detected by a luminometer (Bio-Tek Synergy Neo2, Winooski, VT, USA).

### 2.6. ROS Measurement

The level of intracellular and Total ROS were evaluated by the uptake of H2DCFDA (Invitrogen). To visualize the lipid ROS, cells were seeded in 96-well plates and treated with the designated conditions; then, cells were stained with H2DCFDA following the manufacturer’s instructions. After 30 min in the dark, the cells were detected by a luminometer (Bio-Tek Synergy Neo2, USA).

### 2.7. Proliferation Assay

For the proliferation assays, 5 × 10^3^ cells were seeded into 96-well plate with 100 μL RPMI 1640 supplemented with 10% FBS, using the Cell Counting Kit (CCK8, C0039, Beyotime Biotechnology, Shanghai, China) following the manufacturer’s instructions and detected by a luminometer (MD SpectraMax i3x, Sunnyvale, CA, USA) at 450 nm.

### 2.8. In Vivo Mouse Tumorigenicity Assay

Five-week-old male *BALB/c* nude mice were purchased from Hangzhou Medical College, Hangzhou, China. The mice were housed in individually ventilated cages and supplied with sterilized food, water, and bedding. The mice were maintained at environmental temperature and humidity ranges from 21 to 26 °C and from 50% to 70%, respectively. DLD1-OB, BRAF, BARFV600E, V600E-OB, V600E-OL cells (5 × 10^6^) in 200 μL PBS were inoculated subcutaneously into the right shoulders of *BALB/c* nude mice. Mice were randomly allocated into different groups and intraperitoneally injected with cisplatin (MCE, HY-17394) at a dose of 15 mg/kg or the same amount of saline water for control every other day, until the tumor volume reached 100–150 mm^3^. Tumor length and width were measured with calipers, and tumor volumes were calculated according to the equation: V (mm^3^) = ½ [width2 (mm^2^) × length (mm)]. Growth curves were plotted based on mean tumor volume within each experimental group at the indicated time points. Tumor growth was observed for 15 days. Ethics approval and consent to participate for all human tissues and data and animals involved in this study were approved by the Ethics Committee of the First Affiliated Hospital, Zhejiang University, School of Medicine.

### 2.9. Clinical CRC Specimens, Immunohistochemical Staining (IHC) and Scoring

Through a retrospective search of our pathology database, we ultimately identified 61 cases with BRAF wild-type status and 19 cases harboring the BRAFV600E mutation, which had been confirmed by fluorescence PCR using the human BRAF gene V600E mutation detection kit. These specimens were subsequently processed for immunohistochemical analysis. Immunohistochemical staining by the Envision method was performed on formalin-fixed paraffin-embedded slides, which had been dewaxed and rehydrated before the antigen retrieval step [20]. Each specimen was scored according to the proportion of positive tumor cells: 0, negative or <5%; 1, 5–25%; 2, 26–50%; 3, 51–75%; 4, >75%. The score of staining intensity was kept in range of 0 to 3 based on: 0 for no staining, 1 for weak staining, 2 for moderate staining and, 3 for strong staining. Subsequently, LCN2 (1:200, ab41105), PTGS2 (1:200, AF7003) and cleaved caspase3 (c-caspase3, AF7022, 1:100, Affinity Biosciences, Changzhou, Jiangsu, China) staining were defined as negative (score 0) and positive (scores ≥ 1). LCN2 was defined as low (score < 6) or high (score ≥ 6). Immunohistochemical staining images were from the database in pathology department of the first affiliated hospital, School of Medicine, Zhejiang University. Clinical specimens of all 80 patients were enrolled with informed consent in accordance with a protocol approved by the Ethics Committee of the first affiliated hospital, School of Medicine, Zhejiang University.

### 2.10. Statistical Analysis

All statistical analyses were performed using Prism 8.0 (GraphPad Software) and SPSS (version 24.0; IBM, New York, NY, USA). For results from cell lines, all data are reported as mean ± S.D. Comparisons between two groups were performed using a two-tailed unpaired Student’s *t*-test. Comparisons involving multiple groups or multiple experimental factors were analyzed using two-way analysis of variance (two-way ANOVA), followed by appropriate multiple-comparison tests. Correlation coefficients were calculated by the Spearman rank correlation test and were considered significant at *p* < 0.05.

The FPKM gene expression matrix of COAD obtained from the TCGA database was log-transformed. The expression levels of genes in tumor and normal groups were visualized using boxplots generated with the R package ggplot2. Spearman correlation between two genes was visualized within the tumor and normal groups, respectively. After log transformation of the FPKM matrix, samples from the tumor group were extracted. Patients were stratified into high-expression and low-expression groups based on the median expression level of the gene of interest. Five-year survival analysis was conducted using the R package survival. The geom_signif function was employed to perform Wilcoxon rank-sum tests (wilcox.test) to assess the statistical significance of expression differences between groups.

## 3. Results

### 3.1. BRAF and V600E Mutation Correlate with Ferroptosis Markers in CRC

**Figure 1:**
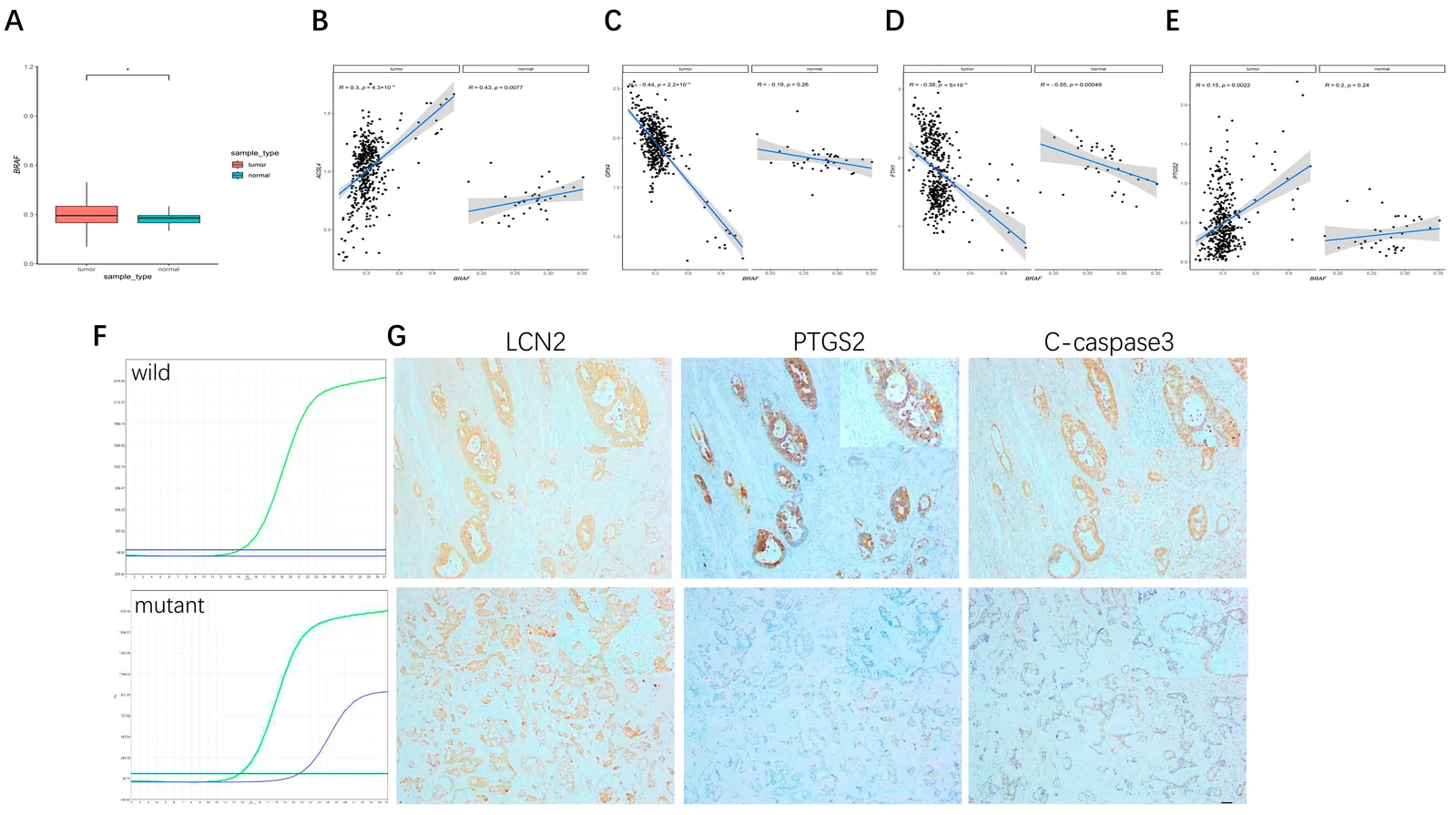
BRAF expression correlates with ferroptosis markers and BRAFV600E mutation is associated with reduced LCN2 and PTGS2 in CRC specimens. (**A**) *BRAF* mRNA expression in CRC and normal tissues from the TCGA database. Correlation analysis between *BRAF* expression and the expression of ferroptosis markers *ACSL4* (**B**), *GPX4* (**C**), *FTH1* (**D**), and *PTGS2* (**E**) in TCGA CRC samples. (**F**) BRAF status was detected in the paraffin sections of CRC specimens by real-time PCR. (**G**) Representative IHC images of LCN2, PTGS2 and C-caspase3 expression in clinical CRC specimens. (left, 50×; inset, 200×). Data are presented as mean ± SD. * *p* < 0.05.

Analysis of The Cancer Genome Atlas (TCGA) data revealed that BRAF expression was significantly elevated in tumors compared to normal tissues (Figure 1A). BRAF expression showed a positive correlation with the pro-ferroptotic markers ACSL4 (Figure 1B) and PTGS2 (Figure 1E) and a significant negative correlation with the anti-ferroptotic molecules GPX4 (Figure 1C) and FTH1 (Figure 1D), suggesting a potential role for BRAF in promoting ferroptosis.

We next selected a cohort of CRC specimens with confirmed BRAFV600E mutation or wild-type status and detected LCN2 and PTGS2 expression. The results showed tumors harboring V600E mutation exhibited significantly lower expression of both LCN2 (57.89% vs. 81.97%) (Figure 1F) and PTGS2 (52.63% vs. 75.41%) (Figure 1G) compared to that of wild-type tissues (Table 1), suggesting the BRAFV600E mutation may be involved in ferroptosis by regulating these related proteins.

### 3.2. BRAF Is Correlated with LCN2 and the V600E/NF-κB/LCN2 Signaling Pathway Is Involved in Cisplatin Induced Ferroptosis

**Figure 2:**
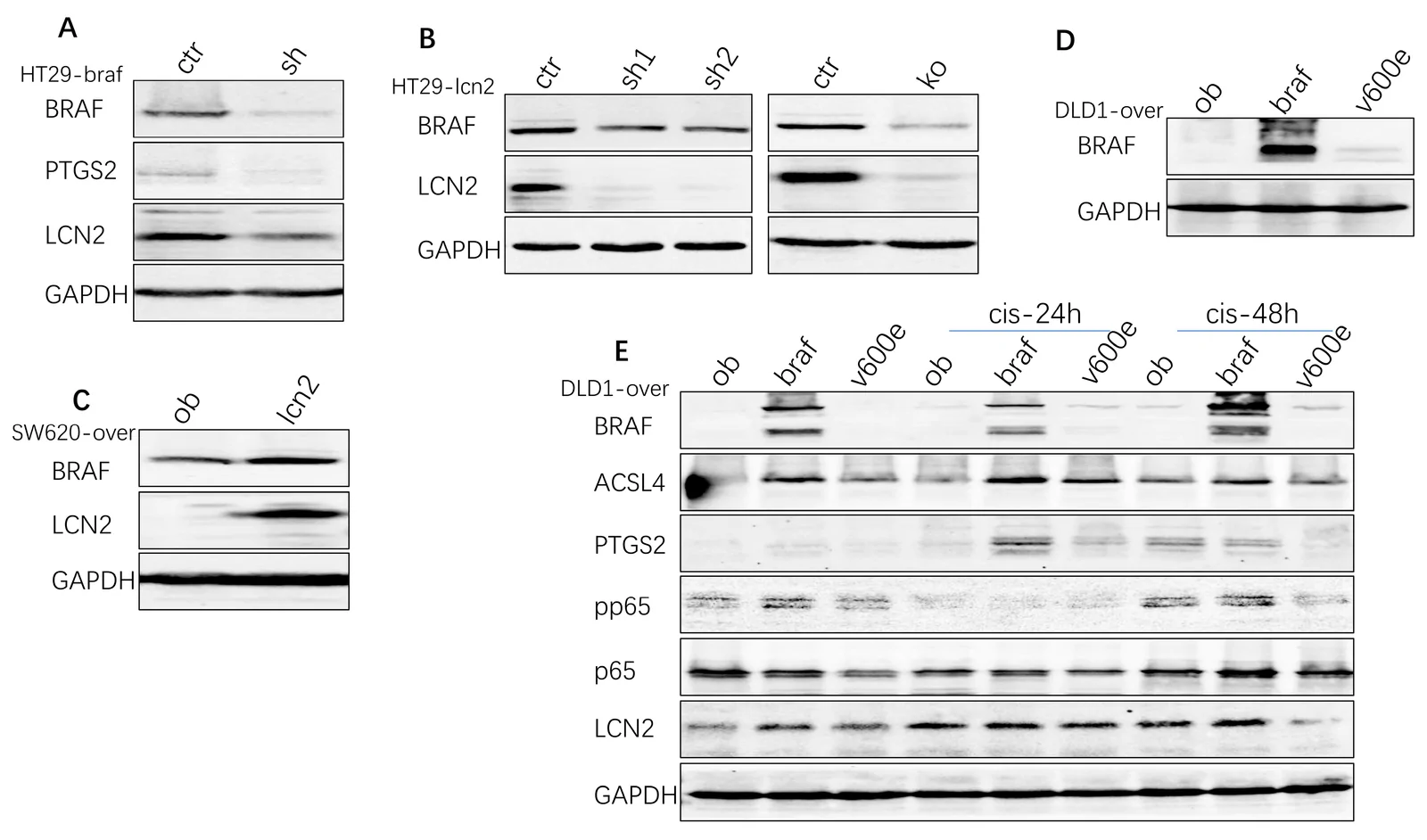
BRAF and LCN2 exhibit a positive correlation in CRC cells. (**A**) Western blot detection of LCN2 and PTGS2 expression in control (shCtrl) and *BRAF*-knockdown (sh*braf*) HT29 cells. (**B**) Expression of BRAF and LCN2 in control (shCtrl), *LCN2*-knockdown (sh*lcn2*), and *LCN2*-knockout (*lcn2*-KO) HT29 cells. (**C**) BRAF expression in control (Vector) and *LCN2*-overexpressing (*lcn2*-OE) SW620 cells. (**D**) Establishment and detection of *BRAF* and V600E overexpression cell lines in DLD1 cell lines. (**E**) Detection of related marker expression after overexpression of *BRAF* and V600E under cisplatin treatment in corresponding cells. The uncropped blots are shown in Supplementary File S1.

To elucidate the relationship between BRAF and LCN2, we established *BRAF*-knockdown and *LCN2*-knockdown/knockout (KO) models in HT29 CRC cells, and *LCN2*-overexpressing SW620 cells. The analysis revealed that *BRAF* knockdown resulted in decreased levels of both LCN2 and PTGS2 (Figure 2A). Interestingly, either knockdown or complete knockout of *LCN2* led to significant downregulation of BRAF expression (Figure 2B), while *LCN2* overexpression increased BRAF levels (Figure 2C), implicating a close correlation between BRAF and LCN2.

### 3.3. The BRAFV600E Overexpression Attenuates Cisplatin-Induced Ferroptosis Through Inhibiting the NF-κB/LCN2 Signaling Pathway

**Figure 3:**
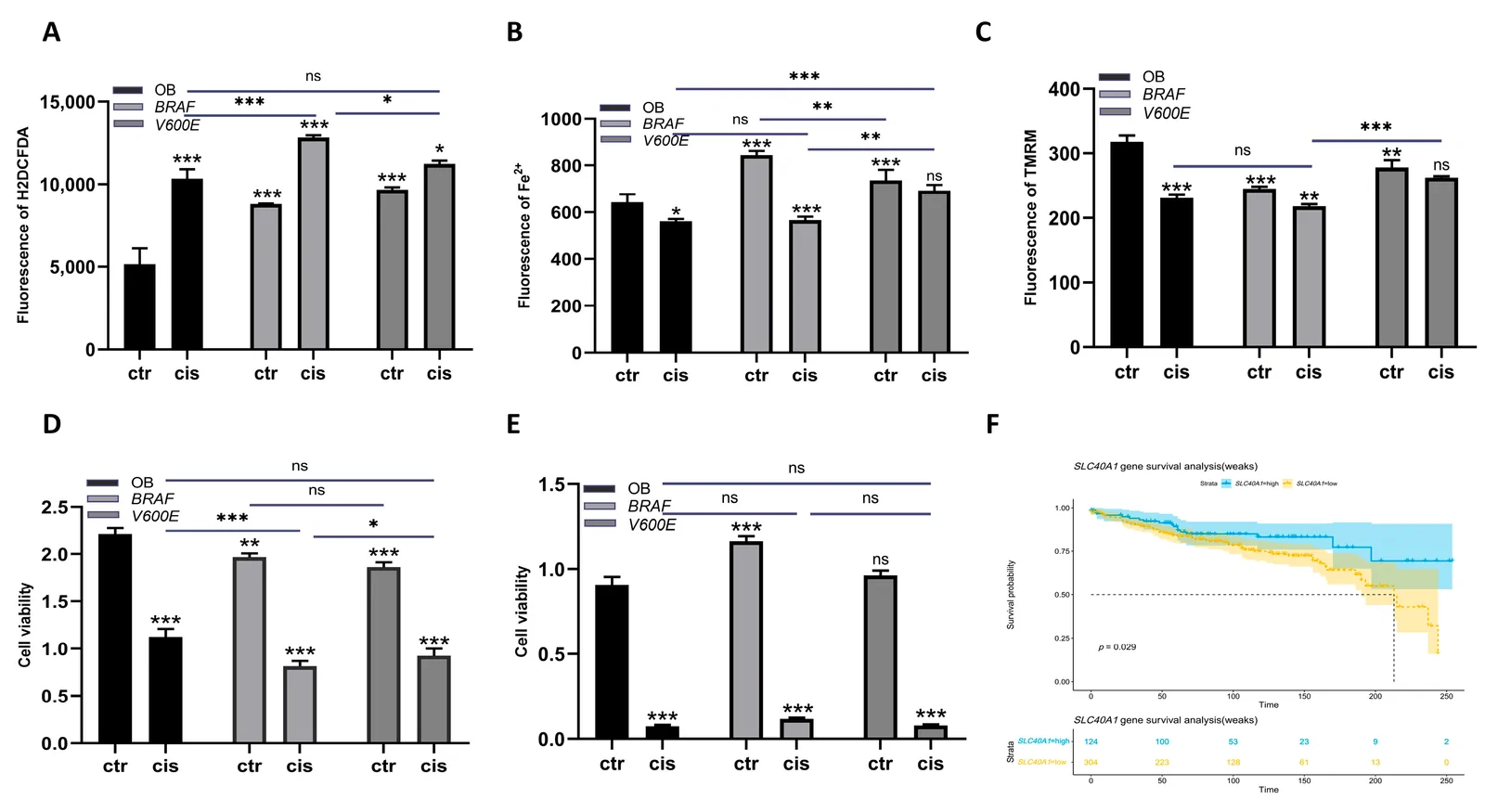
BRAFV600E attenuates the effect of cisplatin on ROS level and cell viability. Intracellular ROS levels (**A**), Fe^2+^ concentrations (**B**) and mitochondrial membrane potential (MMP) levels (**C**) under the treatment of cisplatin for 24 h (30 µM). Cell proliferation detection after 24 h (**D**) and 48 h (**E**) of cisplatin (30 µM) exposure. (**F**) Kaplan–Meier analysis of 5-year survival based on *FPN* (*SLC40A1*) expression in the TCGA CRC cohort. The dashed lines indicate the 50% survival probability and the corresponding median survival time. Data are mean ± SD (**A**–**E**). * *p* < 0.05, ** *p* < 0.01, *** *p* < 0.001; ns, not significant.

To further explore the role BRAFV600E and LCN2 play in cisplatin-induced ferroptosis, we generated DLD1 cells that stably overexpressing *BRAF* or *BRAFV600E* (Figure 2D). The results demonstrate that both *BRAF* overexpression and cisplatin treatment could markedly increase the expression of ACSL4, LCN2 and the phosphorylated p65 level. Elevated expression of PTGS2 was observed after 24 h of cisplatin treatment. Notably, *V600E* overexpression mitigated the effect of cisplatin in *BRAF* overexpression cells (Figure 2E), implying that *V600E* overexpression might be a negative modulator for cisplatin induced ferroptosis through regulating the NF-κB/LCN2 signaling pathway. Subsequent results show that the significantly elevated intracellular ROS levels induced by *BRAF* overexpression and cisplatin treatment were downregulated by the *V600E* overexpression (Figure 3A). *V600E* overexpression also attenuated the *BRAF*-overexpression-induced accumulation in intracellular Fe^2+^ levels (Figure 3B). Interestingly, cisplatin independently decreased cellular iron levels (Figure 3B). Cell proliferation assays indicated *V600E* overexpression conferred a slight protective effect against cisplatin at 24 h, though this effect diminishes significantly over time (Figure 3D,E). Our published data shows *LCN2* knockdown could trigger the expression and activity of ferroportin (FPN), the sole iron exporter. Moreover, the TCGA database analysis shows low FPN expression correlates with worse 5-year survival (Figure 3F).

**Figure 4:**
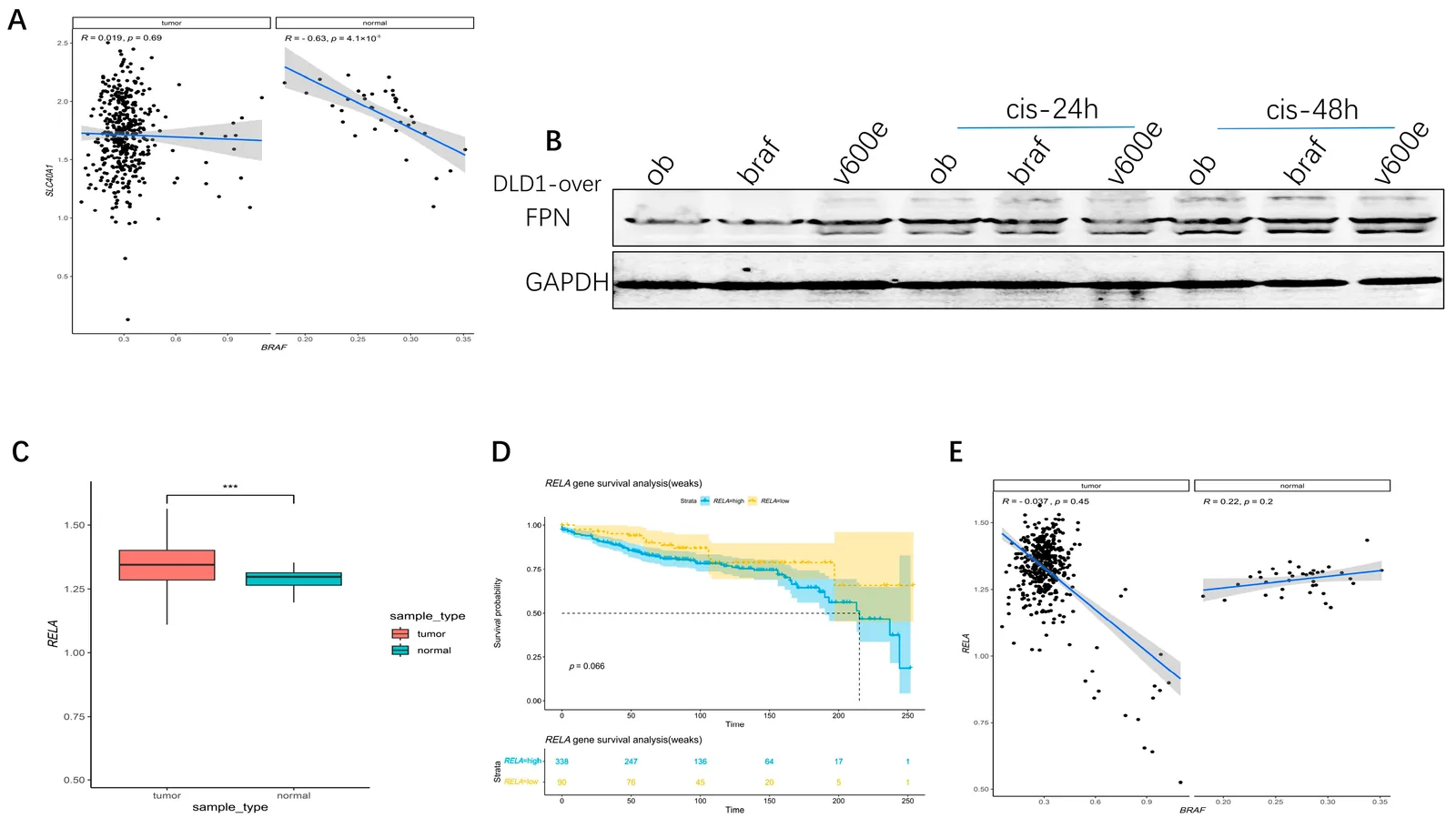
Analysis of BRAF, and FPN and NF-κB p65 (RELA) expression. Correlation between *BRAF* and *FPN* (*SLC40A1*) (**A**) and *RELA* (**E**) mRNA expression in TCGA CRC and normal tissues. (**B**) Western blot analysis of FPN expression in relevant DLD1 cell lines treated with cisplatin (30 µM, 24 h). (**C**) *RELA* mRNA expression was elevated in TCGA CRC specimens compared to normal tissues. (**D**) Correlation between *RELA* expression and 5-year survival rate in the TCGA database. The dashed lines indicate the 50% survival probability and the corresponding median survival time. Data are mean ± SD (**C**). *** *p* < 0.001. The uncropped blots are shown in Supplementary File S1.

Although a significant negative correlation was observed in normal tissues, no obvious correlation was found between *BRAF* and *FPN* (*SLC40A1*) expression in tumors in the TCGA database (Figure 4A). Consistent with iron assay results, western blot analysis revealed that *V600E* overexpression modestly increased FPN expression but did not significantly change the effect of cisplatin (Figure 4B). Collectively, these results suggest that the V600E may confer resistance to cisplatin-mediated ferroptosis.

### 3.4. LCN2 Mitigates Cisplatin Resistance Through the BRAFV600E/NF-κB Pathway

The TCGA analysis shows *NF-ĸB p65* (*RELA*) is overexpressed in tumors (Figure 4C); however, its expression lacked correlation with 5-year survival or *BRAF* levels (Figure 4D,E). Intriguingly, phosphorylated p65 (pp65) and LCN2 levels were markedly reduced in V600E-overexpressing cells after a 48-h cisplatin exposure (Figure 2E).

**Figure 5:**
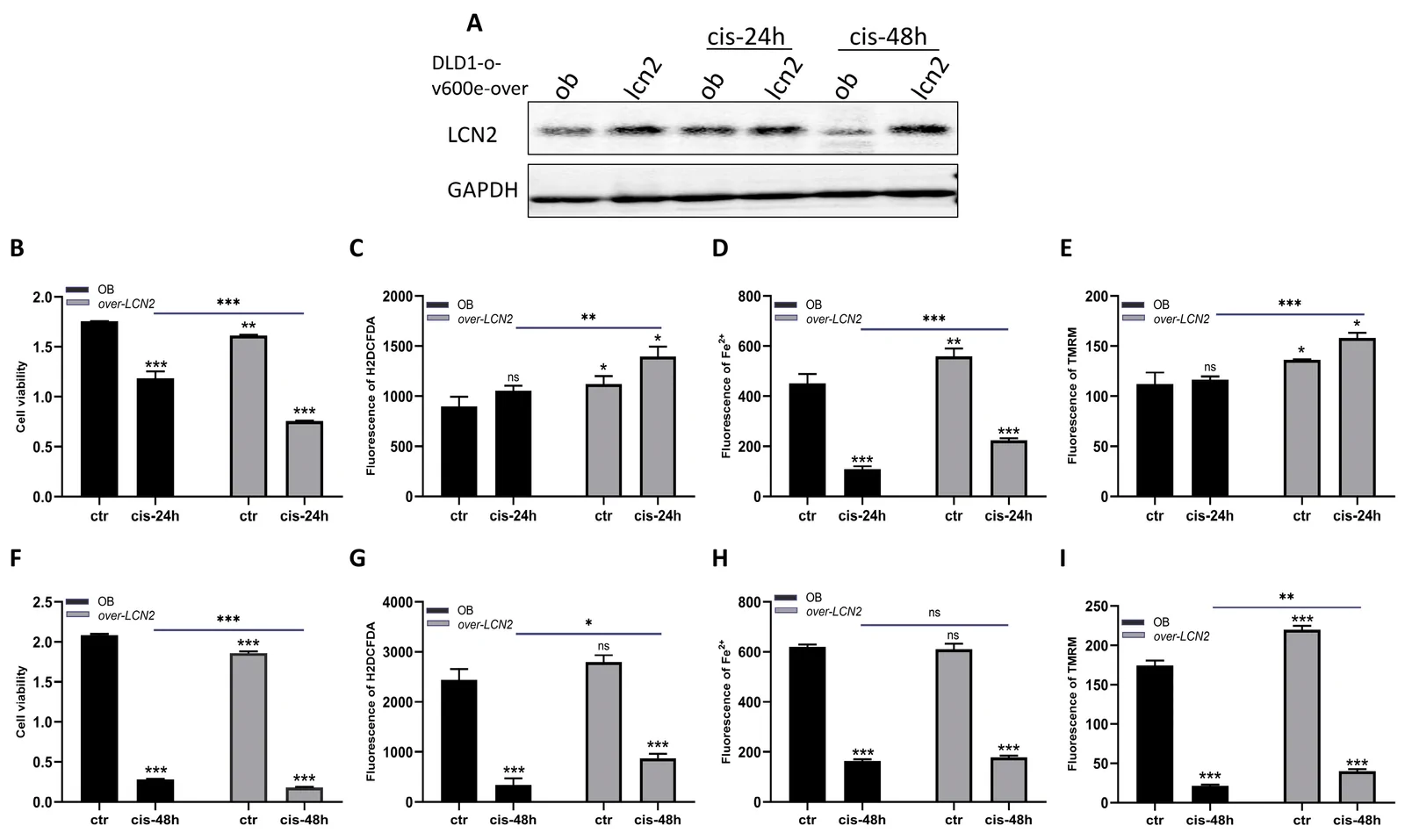
*LCN2* overexpression rescues cisplatin sensitivity in CRC cells expressing *BRAFV600E*. (**A**) Western blot validation of stable *LCN2* expression in the dual overexpression system (*V600E* + *LCN2*-OE). (**B**–**I**) Functional assays in *V600E*-overexpression cells with or without *LCN2* overexpression, following treatment with cisplatin (30 µM) for 24 h (**B**–**E**) and 48 h (**F**–**I**): cell viability (**B**,**F**), intracellular ROS (**C**,**G**), Fe^2+^ levels (**D**,**H**) and mitochondrial membrane potential (MMP) levels (**E**,**I**). Data are mean ± SD (**B**–**I**). * *p* < 0.05, ** *p* < 0.01, *** *p* < 0.001; ns, not significant. The uncropped blots are shown in Supplementary File S1.

To explore the role of LCN2 in V600E/NF-ĸB signaling pathway-regulated ferroptosis under cisplatin treatment, we restored LCN2 expression in *V600E* overexpression cells. The results showed that *LCN2* overexpression significantly augmented cisplatin’s cytotoxic effects on cell viability (Figure 5A,B,F). Furthermore, increased levels of intracellular ROS (Figure 5C,G) and Fe^2+^ (Figure 5D,H) and decreased mitochondrial membrane potential (MMP) levels (Figure 5E,I) were observed in *LCN2* overexpressing cells.

Notably, IHC analysis of CRC specimens revealed a significantly higher proportion of PTGS2-positive cases among LCN2-positive tissues than among LCN2-negative tissues (78.72% vs. 57.58%), and PTGS2 expression correlated positively with LCN2 expression (Table 2). In summary, these results suggest that LCN2 potentiates the sensitivity of CRC cells to cisplatin by promoting V600E/NF-ĸB regulated ferroptosis.

### 3.5. BRAFV600E Overexpression Remarkably Promotes Tumor Growth In Vivo, Whereas Restoration of LCN2 Overcomes Cisplatin Resistance in BRAFV600E Xenograft Models

**Figure 6:**
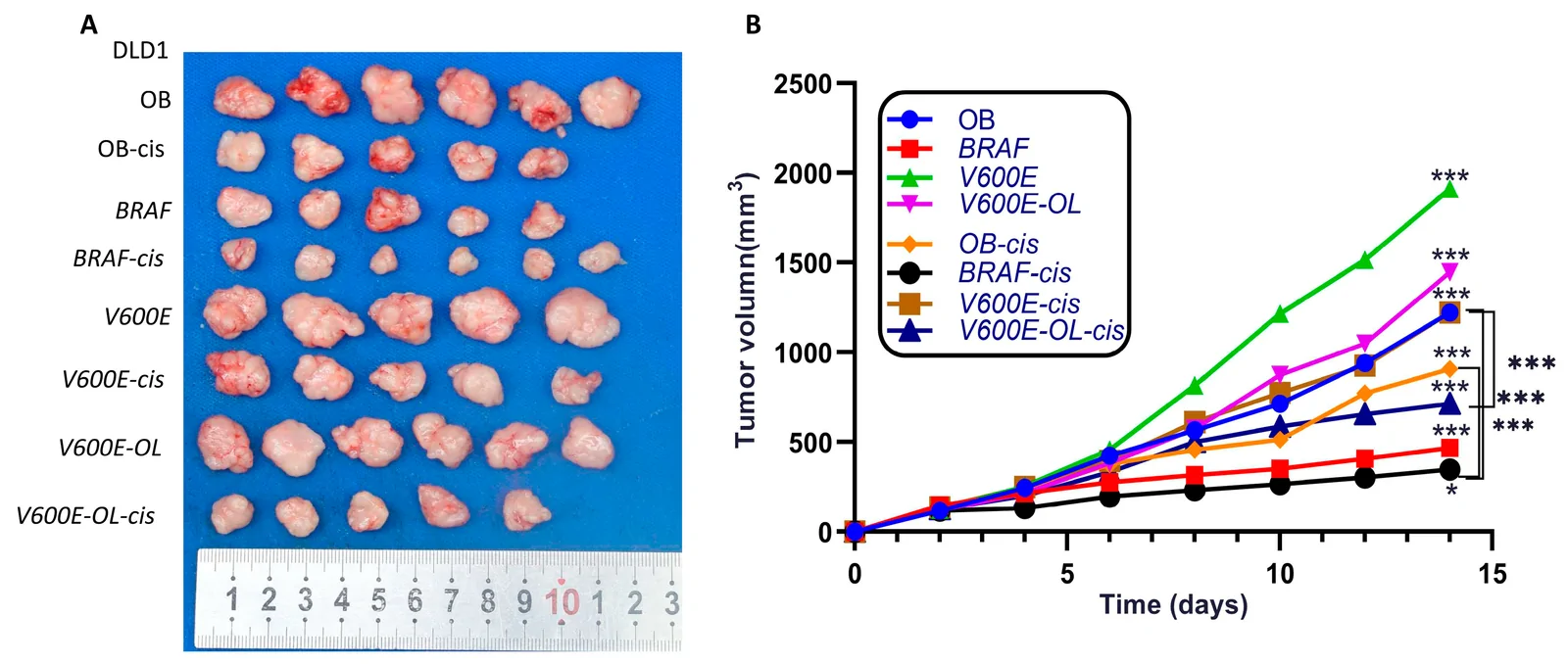
*LCN2* overexpression restores sensitivity in *BRAFV600E*-overexpression-driven xenografts to cisplatin. Representative images (**A**) and tumor volumes (**B**) of subcutaneous tumors from *BALB/c* nude mice in different treatment groups. Control (Vector), *V600E* overexpression (*V600E*-OE), *V600E* + *LCN2* overexpression (*V600E* + *LCN2*-OE), with or without cisplatin treatment. Data are mean ± SD. * *p* < 0.05, *** *p* < 0.001 (comparison between *V600E*-OE + Cisplatin vs. *V600E* + *LCN2*-OE + Cisplatin groups at endpoint).

Further investigation revealed that, with or without cisplatin treatment, tumors overexpressing *BRAFV600E* exhibited significantly larger volumes than those overexpressing wild-type *BRAF*, confirming its role in promoting cisplatin resistance. Notably, tumors co-overexpressing *LCN2* and *BRAFV600E* showed significant growth inhibition in response to cisplatin (Figure 6A,B), implying that LCN2 functions as a critical effector mediating cisplatin resistance conferred by *BRAFV600E* overexpression.

### 3.6. Apoptotic Signaling Contributes to LCN2-Modulated Ferroptosis in BRAFV600E Overexpression Cells

**Figure 7:**
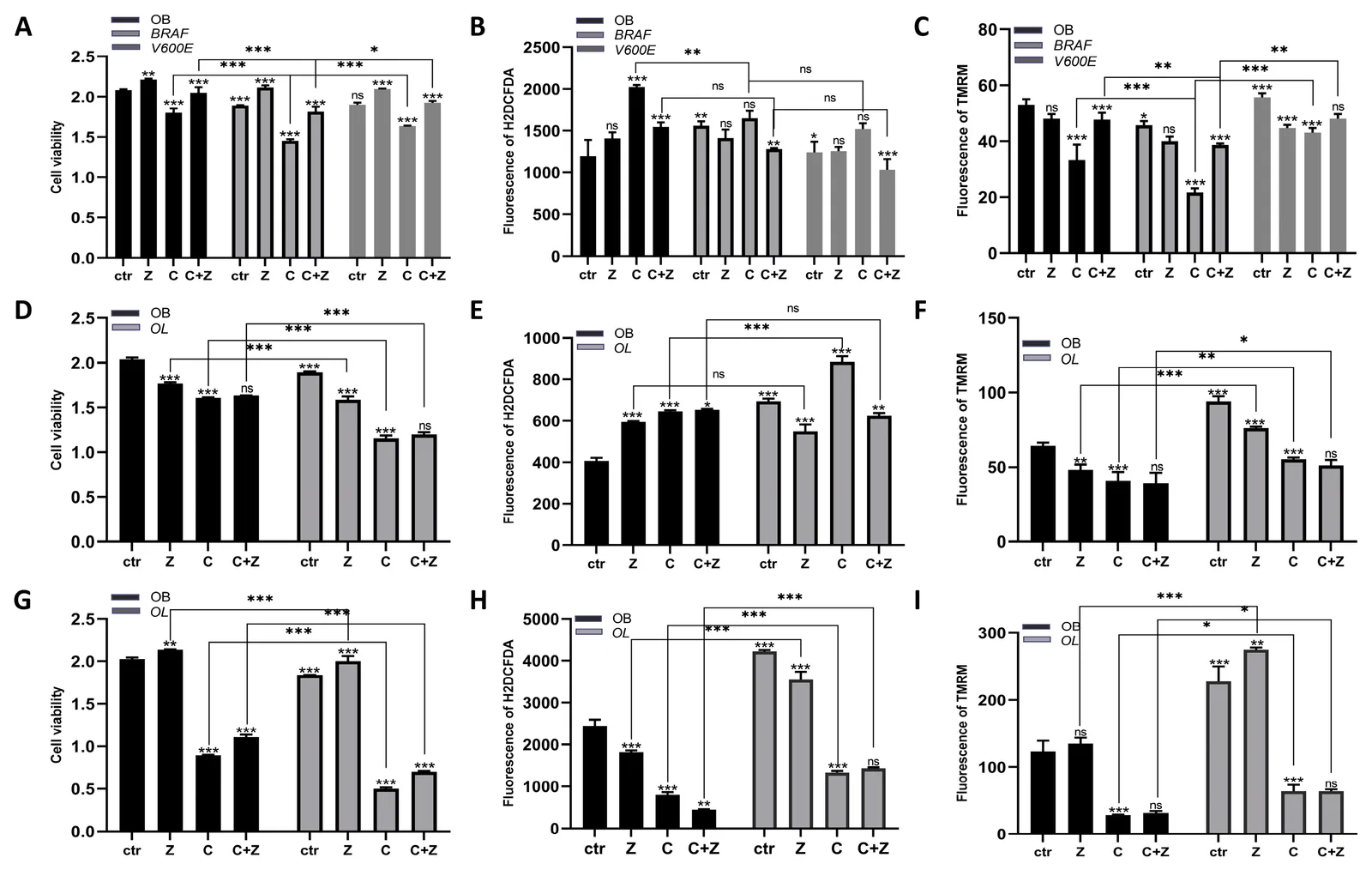
Apoptosis contributes to LCN2-mediated ferroptosis of *V600E* overexpression cells under cisplatin treatment. Cell proliferation capacity (**A**,**D**,**G**), intracellular ROS levels (**B**,**E**,**H**), and mitochondrial membrane potential levels (**C**,**F**,**I**) were detected in the corresponding cells (cisplatin/ZVAD-FMK, 30 μM for 24 h (**A**–**F**) and 48 h (**G**–**I**). Mean ± SD, * *p* < 0.05, ** *p* < 0.01, *** *p* < 0.001, ns, not significant).

It was noted that cisplatin treatment could reduce MMP levels, a hallmark of apoptosis, whereas *V600E* overexpression reversed the effect of cisplatin (Figure 3C), suggesting that apoptosis might be involved in *BRAFV600E*-overexpression-modulated cisplatin-induced ferroptosis. Subsequently analysis found that inhibition of apoptosis by pan-caspase inhibitor ZVAD-FMK (Figure 7C) could promote the survival ability of *BRAF/V600E* overexpression cells to cisplatin treatment (Figure 7A) and markedly inhibit the increased levels of ROS induced by cisplatin and *BRAF* overexpression (Figure 7B). Further investigation showed that *LCN2* overexpression could sensitize *BRAFV600E*-overexpressing cells to treatment with cisplatin, with or without ZVAD-FMK (Figure 7D,G), as evidenced by reduced cell viability and increased ROS accumulation (Figure 7E,H) and MMP levels (Figure 7F,I), though there was no obvious change of ROS and MMP levels between cisplatin and cisplatin combined with ZVAD-FMK after 48 h treatment (Figure 7H,I).

**Figure 8:**
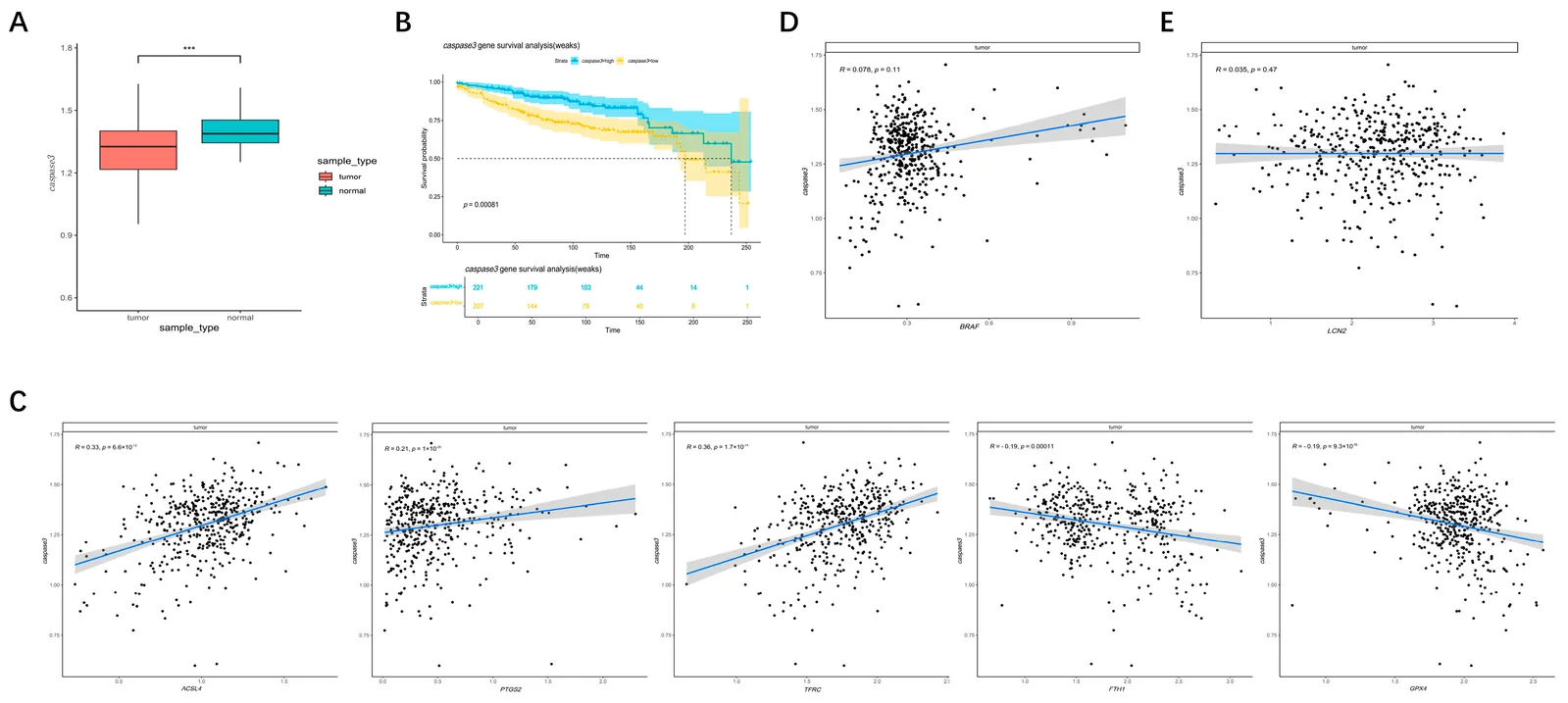
Correlation analysis of ferroptosis and apoptosis markers in CRC tissues from the TCGA database. (**A**) *Caspase3* expression in CRC. (**B**) 5-year survival rate based on *caspase3* expression. (**C**) Correlation between *caspase3* expression and ferroptosis-related markers (*ACSL4*, *PTGS2*, *TFRC*, *FTH1*, and *GPX4*). (**D**,**E**) Correlation analysis of *BRAF* expression with *LCN2* (**D**) and *caspase3* (**E**). *** *p* < 0.001.

Analysis of the TCGA database revealed that the critical molecular marker of apoptosis, *caspase3*, exhibited significantly reduced expression in tumors (Figure 8A). Moreover, its low expression was associated with markedly decreased 5-year survival rates (Figure 8B). Further analysis demonstrated positive correlations between *caspase3* and ferroptosis markers *ACSL4*, *PTGS2*, and *TFRC*, while showing negative relationships with *FTH1* and *GPX4* (Figure 8C), suggesting a potential link between apoptosis and ferroptosis. Additionally, immunohistochemistry staining results from CRC samples revealed a positive correlation between cleaved-caspase3 and PTGS2 expression (Table 3), with significantly higher PTGS2 positivity rates in cases positive for cleaved-caspase3 (89.47% vs. 63.93%). Cleaved-caspase3 expression showed lower expression in the V600E mutation group than in the wild-type group (Figure 1D), though the difference did not reach statistical significance. Regrettably, the TCGA data analysis failed to identify any association between *BRAF* or *LCN2* and *caspase3* (Figure 8D,E).

In conclusion, these results suggest that apoptosis inhibition might contribute to cisplatin resistance via the suppression of ferroptosis in association with BRAFV600E overexpression and the NF-κB/LCN2 axis within this experimental context.

## 4. Discussion

BRAFV600E-mutant metastatic CRC (mCRC), represents a molecular subtype characterized by aggressive biology, frequent right-sided and MSI-associated clinicopathologic features, and poor outcomes after conventional systemic therapy, with a median overall survival of 4–6 months after failure of initial therapy [3,7]. Advances of chemotherapies have been obtained in recent years. Research shows that a combination of encorafenib, cetuximab, and binimetinib resulted in significantly longer overall survival and a higher response rate than standard therapy in patients with mCRC harboring the BRAF V600E mutation [32]. More recently, a study revealed that first-line treatment with encorafenib plus cetuximab (EC)+mFOLFOX6 has a significantly longer progression-free survival (median, 12.8 vs. 7.1 months) and overall survival (median, 30.3 vs. 15.1 months) than with standard care [33]. However, these advances do not eliminate the problem of intrinsic or acquired chemotherapy resistance. Instead, they emphasize the need to understand how BRAFV600E manipulates DNA-damage responses, redox homeostasis, and regulated cell death programs across different combination of chemotherapies. Our results show that dysregulation of the established NF-κB/LCN2 signaling axis is associated with ferroptosis resistance and decreased cisplatin sensitivity in BRAFV600E-mutant colorectal cancer.

Studies have shown the activated NF-κB signaling pathway regulates the expression of various genes associated with invasion and drug resistance in BRAFV600E-mutated thyroid cancer [34]. Moreover, the NF-κB/LCN2 axis has been implicated in chemoresistance by modulating the tumor microenvironment and cell survival [35] and enhancing the resistance of CRC cells to 5-FU [36]. However, this study suggests that suppression of NF-κB/LCN2 signaling pathway leads to insensitivity of BRAFV600E-overexpressing CRC cells to cisplatin treatment. The current study focused primarily on cisplatin-induced biological responses rather than the basal signaling differences between BRAF wild-type and BRAFV600E overexpression cells. Therefore, whether BRAFV600E directly suppresses NF-κB/LCN2 signaling under basal conditions or predominantly attenuates its activation following cisplatin treatment warrants further investigation. Further functional studies using pharmacological or genetic modulation of NF-κB activity would provide additional mechanistic insight into the regulation of the NF-κB/LCN2 signaling axis in BRAFV600E-mutant colorectal cancer.

LCN2 is identified as a core factor in iron metabolism [9]. Ferroptosis is a type of cell death that depends on iron; accumulation of iron stimulates ROS formation via the Fenton reaction, thereby initiating lipid peroxidation [37,38]. Nevertheless, the role of LCN2 in cisplatin-associated ferroptosis remains controversial, and accumulating evidence suggests that its biological function may be highly dependent on tumor type, genetic background, and cellular context.

On the one hand, reduced LCN2 expression could ameliorate erastin-mediated ferroptosis, meanwhile, increase cisplatin vulnerability in breast cancer cells [29], and promote cisplatin sensitivity of oral squamous cell carcinoma [39]. Furthermore, knockdown of LCN2 could lead to ferroptosis and enhance sensitivity of lung cancer cells to anti-PD-L1 therapy [40]. Interestingly, Chaudhary et al. demonstrated that LCN2 promoted CRC progression and chemoresistance of 5-fluorouracil by suppressing ferroptosis through activation of GPX4/xCT signaling and reduction of intracellular iron levels [41]. On the other hand, researches revealed that LCN2 acts as a biomarker of cisplatin-induced acute kidney injury or nephrotoxicity [42,43], and increased levels of LCN2 could protect the kidney cells against cisplatin-induced injury [44]. In addition, knockdown of LCN2 significantly promotes the chemoresistance in oral cancer cell [45]. Collectively, these findings indicate that the biological function of LCN2 during cisplatin treatment is highly context-dependent and may differ substantially among tissues and disease models.

Our results showed that LCN2 expression was reduced in BRAFV600E-overexpessing CRC cells. Restoration of LCN2 expression could partially enhance intracellular iron accumulation, promote the iron-dependent Fenton reaction, increase ROS levels, and subsequently increase cisplatin vulnerability both in vitro and in vivo. Consistent with our experimental observations, primary CRC tissues harboring BRAFV600E mutations exhibited lower expression of LCN2 and PTGS2, while analyses of the TCGA datasets further supported a significant association between LCN2 expression and ferroptosis-related markers. In our study, PTGS2 expression was evaluated in primary tissues to maintain consistency with our in vitro mechanistic findings, where it served as a downstream marker of the ferroptosis process. However, it should be noted that PTGS2 expression is not entirely specific to ferroptosis. Accordingly, these findings should be interpreted as complementary evidence supporting the potential involvement of LCN2-mediated ferroptosis in BRAFV600E-associated cisplatin resistance.

The apparent inconsistency between our findings and those reported by Chaudhary et al. deserves particular consideration. Although both studies were performed in CRC, important differences exist in experimental design and biological context. The present study employed an isogenic BRAFV600E overexpression model specifically established to investigate BRAFV600E-overexpression-associated cisplatin resistance, whereas the study by Chaudhary et al. primarily examined the general role of LCN2 in CRC progression, ferroptosis and chemoresistance of 5-fluorouracil [41]. These differences suggest that the biological function of LCN2 may be influenced by oncogenic driver mutations, cellular iron metabolism, and treatment-related stress. Taken together, our results support the hypothesis that restoration of LCN2 may enhance ferroptosis susceptibility through regulation of iron homeostasis in the specific context of BRAFV600E-overexpression-associated cisplatin resistance. Accordingly, our findings should not be interpreted as contradicting previous observations, but rather as supporting a context-dependent roe of LCN2 in ferroptosis regulation under specific genetic and therapeutic conditions.

Previous study has revealed seven upregulated and seven downregulated apoptosis correlated proteins in the liver of LCN2-knockout mice [30]. Moreover, secreted LCN2 regulated by IL-3 could induce apoptosis [46]. Interestingly, LCN2 could enhance cardiomyocyte apoptosis through increasing intracellular iron accumulation [47], suggesting the crosstalk between ferroptosis and apoptosis. Previous studies have also implicated the involvement of BRAFV600E in the regulation of apoptosis in both CRC [48] and thyroid cancer [49]. Intriguingly, our study revealed that the accumulation of ROS and suppressed cell viability induced by cisplatin could be reversed by either V600E overexpression or inhibition of apoptosis by ZVAD-FMK treatment. Notably, restored expression of LCN2 in V600E-overexpression cells could alleviate the effect of ZVAD-FMK, implying the involvement of apoptosis in V600E/LCN2 signaling regulated ferroptosis under cisplatin treatment. Although these findings suggest that apoptosis might involve in LCN2-regulated ferroptosis during cisplatin treatment, further mechanistic exploration is required to fully elucidate this interaction.

Beyond its role in iron metabolism, the NF-κB/LCN2 axis may be involved in a broader network of redox regulation that influences cisplatin resistance in BRAFV600E-mutant CRC. Our data show that suppressing of the NF-κB/LCN2 axis was associated with reduced intracellular Fe^2+^ accumulation, attenuated ferroptosis, leading to decreased sensitivity to cisplatin. These observations align with previous findings that demonstrated that oncogenic KRAS promotes chemoresistance by activating NRF2-dependent metabolic adaptation and antioxidant defense [50], whereas inhibiting of the SIRT1/NRF2 pathway restores cisplatin sensitivity by enhancing ferroptosis in CRC [51]. Although these studies mainly focused on antioxidant defense rather than iron metabolism, their findings are consistent with our results, supporting the hypothesis that disruption of redox homeostasis, either through altered antioxidant capacity or impaired iron-dependent oxidative stress, may contribute to chemotherapy resistance. Whether NRF2 functions independently of the NF-κB/LCN2 pathway or whether these signaling pathways interact during regulation of ferroptosis remains unclear. Given that NRF2 primarily regulates antioxidant defense, whereas our data suggest that the NF-κB/LCN2 axis influences iron homeostasis, these pathways may represent complementary mechanisms modulating cellular redox homeostasis during cisplatin treatment. Further studies will be required to determine whether functional crosstalk exists between these signaling networks in BRAFV600E-mutant CRC.

Several limitations and experimental considerations of the present study should be acknowledged. First, our research primarily focused on the role of LCN2 in regulating iron homeostasis during cisplatin-induced ferroptosis, rather than providing a comprehensive characterization of the ferroptosis signaling network. Consequently, more comprehensive, ferroptosis-specific validations are warranted in future studies to fully elucidate the downstream mechanisms. Second, our current mechanistic findings were generated using an isogenic DLD1 colorectal cancer model, which harbors an endogenous KRAS mutation. Constitutive KRAS activation may potentially influence downstream signaling events and therefore complicate interpretation of BRAF-dependent biological responses. Although we deliberately employed an isogenic overexpression strategy to minimize genetic heterogeneity and allow direct comparison between BRAF wild-type and BRAFV600E, validation in additional CRC cell lines lacking endogenous KRAS mutations would further support the broader applicability of our findings. Finally, the relatively limited number of BRAFV600E-mutant clinical cases and the reliance on PTGS2 as a single tissue marker represent limitations of the clinical correlation analyses. Although statistically significant associations were observed, these preliminary clinical findings should be interpreted with appropriate caution and require validation in larger, independent patient cohorts using a broader panel of ferroptosis markers.

Collectively, our study proposes that dysregulated ferroptosis regulated by the NF-κB/LCN2 axis contributes to BRAFV600E-associated cisplatin resistance, and apoptosis is involved in the process of cisplatin induced ferroptosis. These findings suggest LCN2 as a potential biomarker to improve therapeutic responses in BRAFV600E-mutant CRC. Although further exploration is needed to elucidate the role of LCN2 in apoptosis progression under cisplatin treatment and the crosstalk between apoptosis and ferroptosis in chemotherapy resistance, this interaction highlights the potential of therapeutic strategies that simultaneously activate ferroptosis and apoptosis to overcome multidrug resistance for this high-risk molecular subtype of CRC.

## 5. Conclusions

In conclusion, this study shows that within a KRAS-mutant CRC background, *BRAFV600E* overexpression and the concomitant dysregulation of the NF-ĸB/LCN2 signaling pathway are associated with impaired iron homeostasis and reduced ferroptosis, thereby contributing to cisplatin resistance in an isogenic model of *BRAFV600E* overexpression. Interestingly, our findings suggest that cisplatin could activate both ferroptosis and apoptosis, inhibition of apoptosis could attenuate cisplatin cytotoxicity on *BRAFV600E*-overexpressing CRC cells, whereas restoration of the expression of *LCN2* could mitigate resistance to cisplatin. Although further validations in KRAS wild-type backgrounds are needed to fully isolate BRAFV600E-specific effects and elucidate the interaction between ferroptosis and apoptosis, this study provides novel insights into the potential of combining induction of ferroptosis and apoptosis to overcome therapeutic resistance, highlighting LCN2 as a promising biomarker and therapeutic target in CRC therapeutic resistance.

## Figures and Tables

**Table 1:** Expression analysis and correlation study of LCN2 and PTGS2 in BRAF wild-type and V600E mutant specimens of colorectal cancer.

IHC		Wild Type (%)	Mutant Type (V600E) (%)	*n*	*p* Value	R Value
LCN2	+	50 (81.97)	11 (57.89)	61	0.031	−0.241
	−	11 (18.03)	8 (42.11)	19		
Total		61	19	80		
PTGS2	+	46 (75.41)	10 (52.63)	56	0.058	−0.212
	−	15 (24.59)	9 (47.37)	24		
Total		61	19	80		

**Table 2:** Expression analysis and correlation study of LCN2 and PTGS2 in BRAF wild-type and V600E mutant groups of colorectal cancer.

IHC		LCN2 (%)		*n*	*p* Value	R Value
		−	+			
PTGS2	−	14 (42.42)	10 (21.28)	24	0.042	0.227
	+	19 (57.58)	37 (78.72)	56		
Total		33	47	80		
Cleaved caspase3	−	5 (26.32)	4 (6.56)	9	0.017	0.266
	+	14 (73.68)	57 (93.44)	71		
Total		19	61	80		

**Table 3:** Correlation between cleaved caspase3 and PTGS2 expression in clinical CRC Tissues.

IHC		Cleaved Caspase3 (%)		*n*	*p* Value	R Value
		−	+			
PTGS2	−	22 (36.07)	2 (10.53)	24	0.034	0.237
	+	39 (63.93)	17 (89.47)	56		
Total		61	19	80		

## Data Availability

The datasets and clinical data utilized in this study are available from the corresponding author on reasonable request.

## Supplementary Materials

The following supporting information can be downloaded at: https://www.mdpi.com/article/10.3390/cancers18162552/s1, Supplementary File S1: Original Image of Western Blot.

## Abbreviations

The following abbreviations are used in this manuscript:

CRC	colorectal cancer
LCN2	Lipocalin2
cis	cisplatin
NF-κB	nuclear factor-kappa B
p65	nuclear factor kappa-B p65
p-p65	phosphorylated nuclear factor kappa-Bp65
FPN	ferroportin
TMRM	Tetramethylrhodamine, mitochondrial membrane potential (MMP) indicator
Ctr	blank control
SHB/OB	blank control of shRNA/overexpression vector
shLCN2	shRNA vector of LCN2
LCN2-KO	knockout vector of LCN2
