# Supplementary material for: NF-κB/Lipocalin 2 Signaling Pathway Mitigates the Chemoresistance of BRAFV600E-Mutant Colorectal Cancer to Cisplatin by Promoting Ferroptosis
Source: Cancers (Basel). 2026 Aug 9;18(16):2552. doi: 10.3390/cancers18162552 (PMC13510473; doi:10.3390/cancers18162552)
Supplement: Supplementary file 1 [file cancers-18-02552-s001.zip › cancers-4443395-supplementary.pdf]

FIGURE 2A

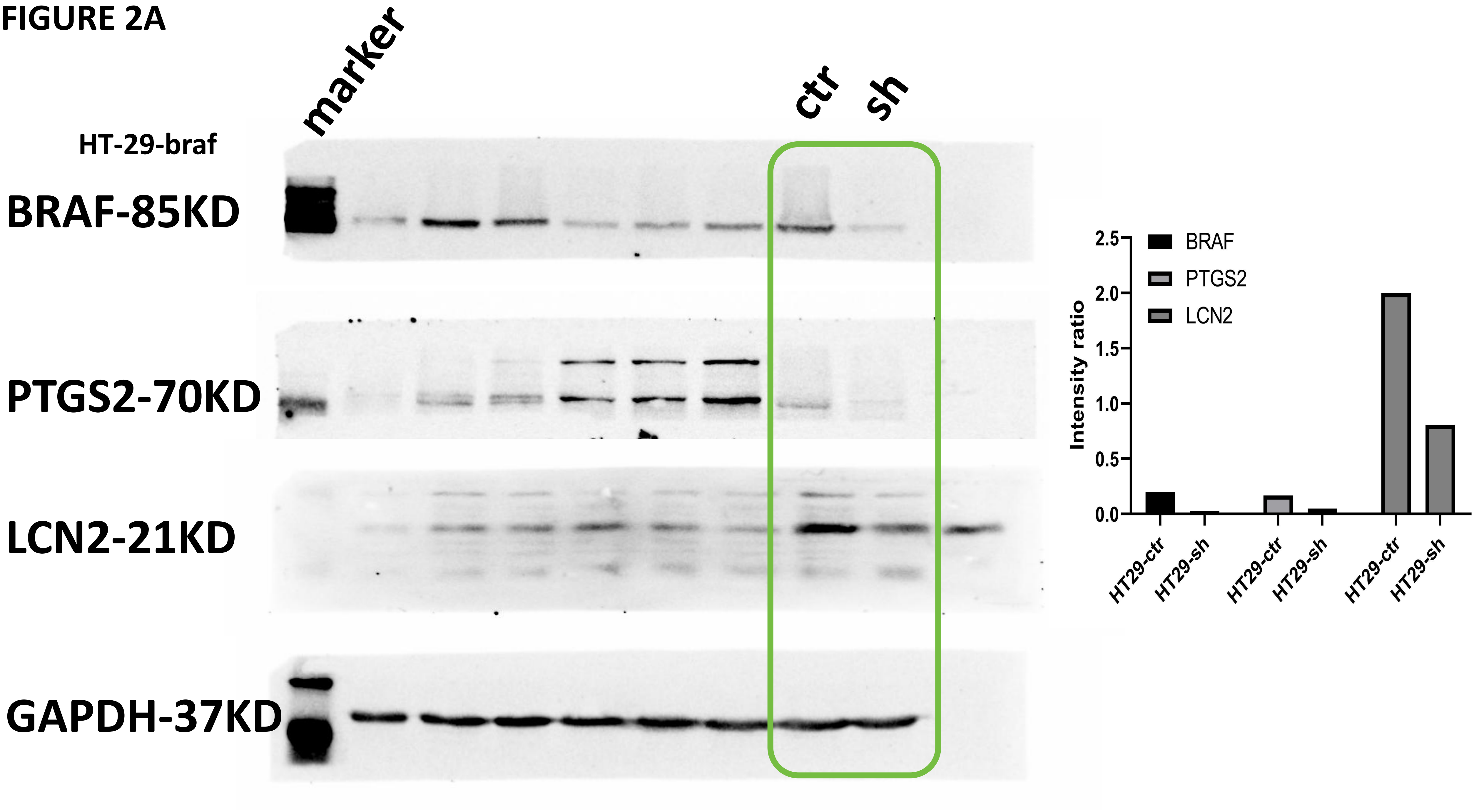
marker
ctr
sh
HT-29-braf
BRAF-85KD
PTGS2-70KD
LCN2-21KD
GAPDH-37KD
Intensity ratio
2.5
2.0
1.5
1.0
0.5
0.0
BRAF
PTGS2
LCN2
HT29-ctr
HT29-sh
HT29-ctr
HT29-sh
HT29-ctr
HT29-sh

FIGURE 2B

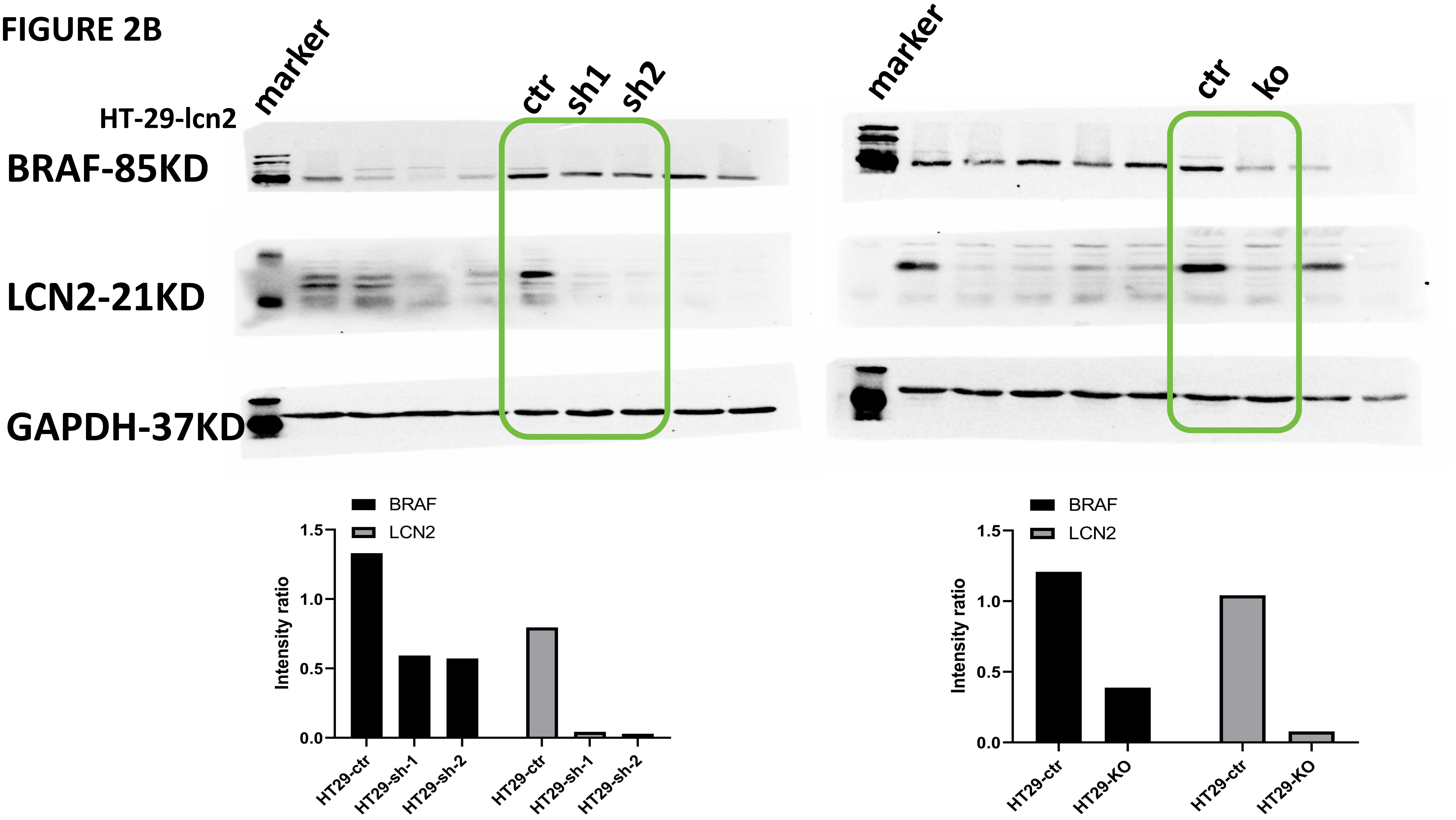

HT-29-lcn2
marker
ctr
sh1
sh2
marker
ctr
ko
BRAF-85KD
LCN2-21KD
GAPDH-37KD
BRAF
LCN2
Intensity ratio
1.5
1.0
0.5
0.0
HT29-ctr
HT29-sh-1
HT29-sh-2
HT29-ctr
HT29-sh-1
HT29-sh-2
BRAF
LCN2
Intensity ratio
1.5
1.0
0.5
0.0
HT29-ctr
HT29-KO
HT29-ctr
HT29-KO

FIGURE 2C

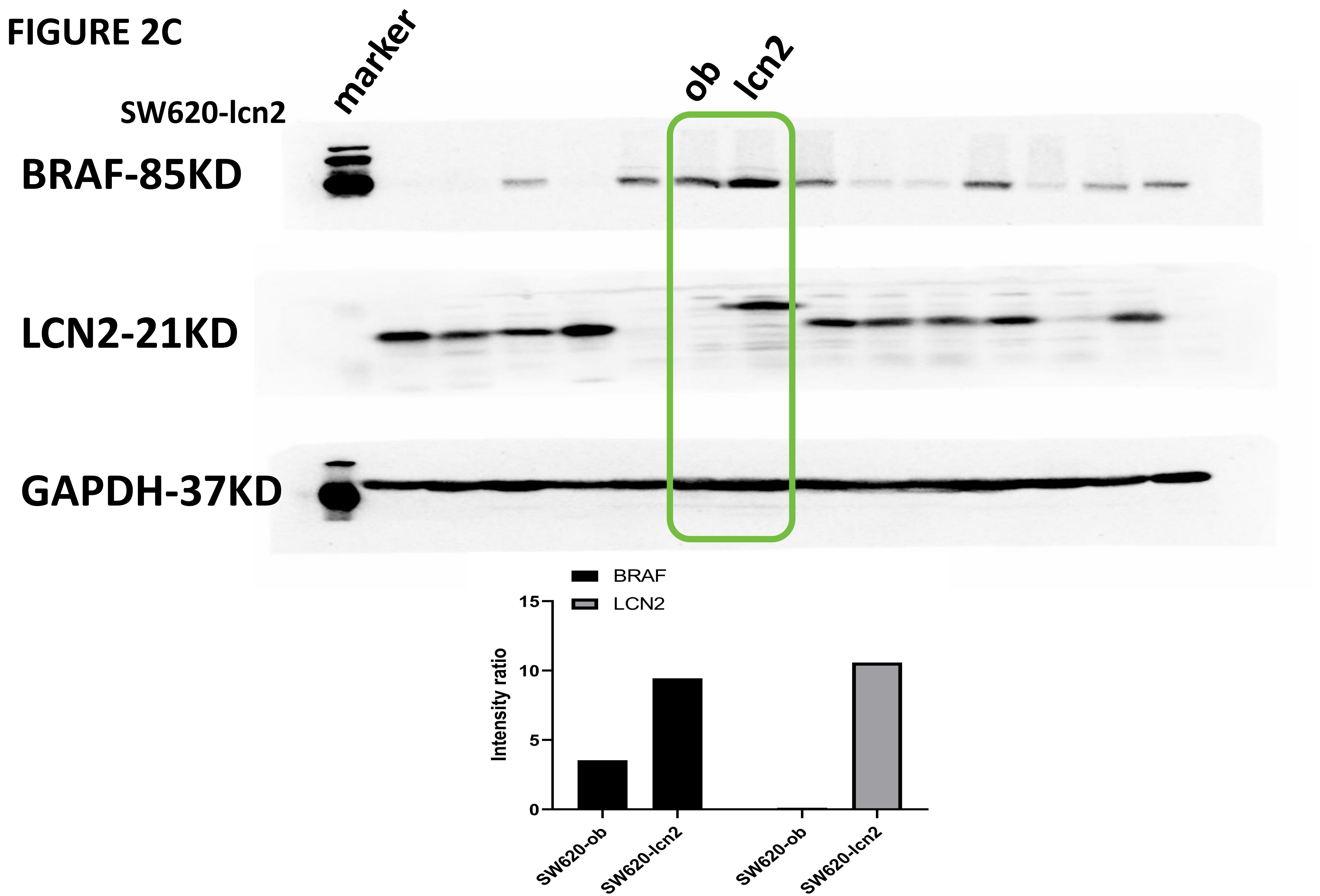

marker
ob
lcn2
SW620-lcn2
BRAF-85KD
LCN2-21KD
GAPDH-37KD
BRAF
LCN2
Intensity ratio
15
10
5
0
SW620-ob
SW620-lcn2
SW620-ob
SW620-lcn2

FIGURE 2D

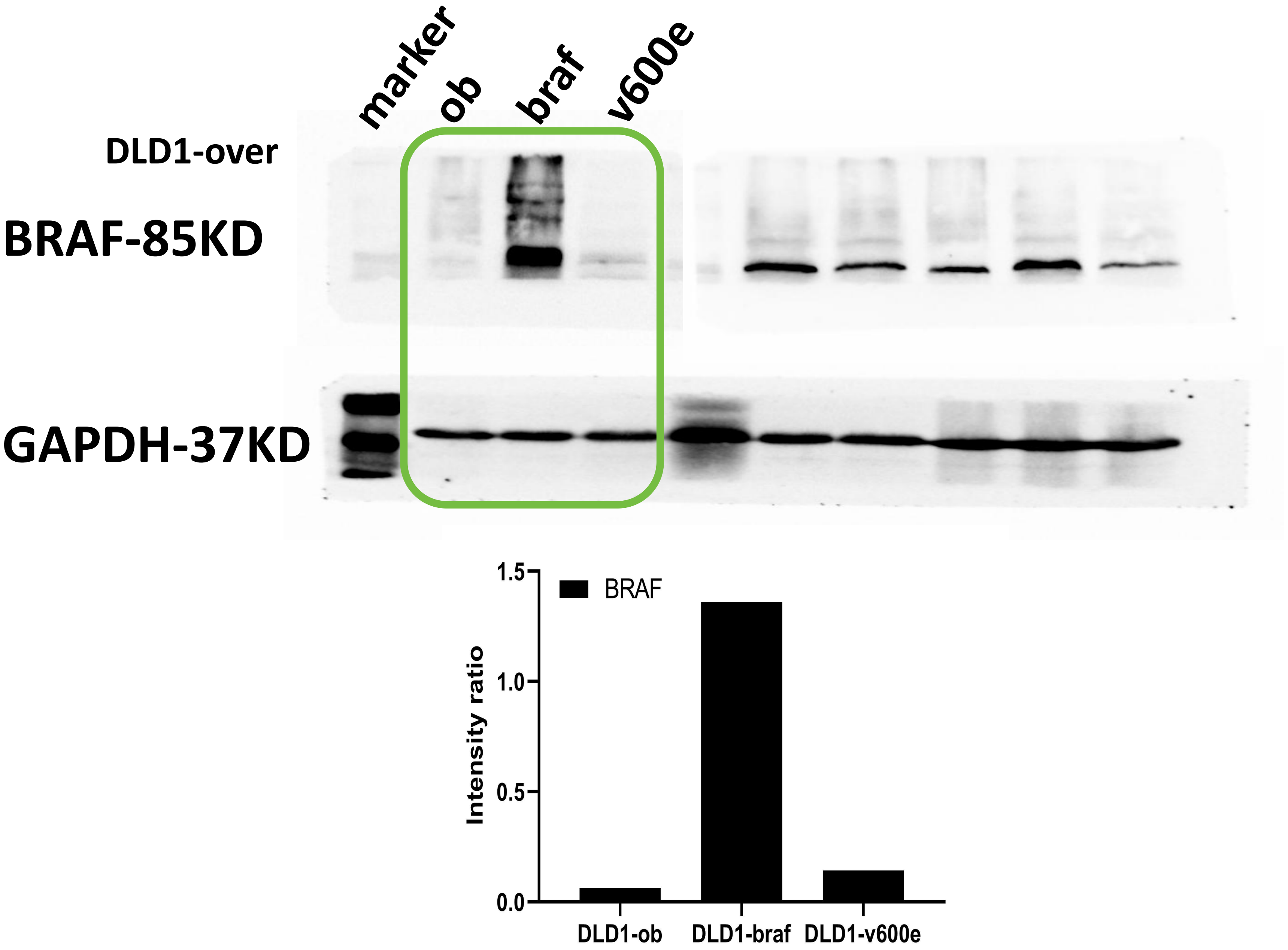

marker
ob
braf
v600e
DLD1-over
BRAF-85KD
GAPDH-37KD
BRAF
Intensity ratio
1.5
1.0
0.5
0.0
DLD1-ob
DLD1-braf
DLD1-v600e

FIGURE 2E

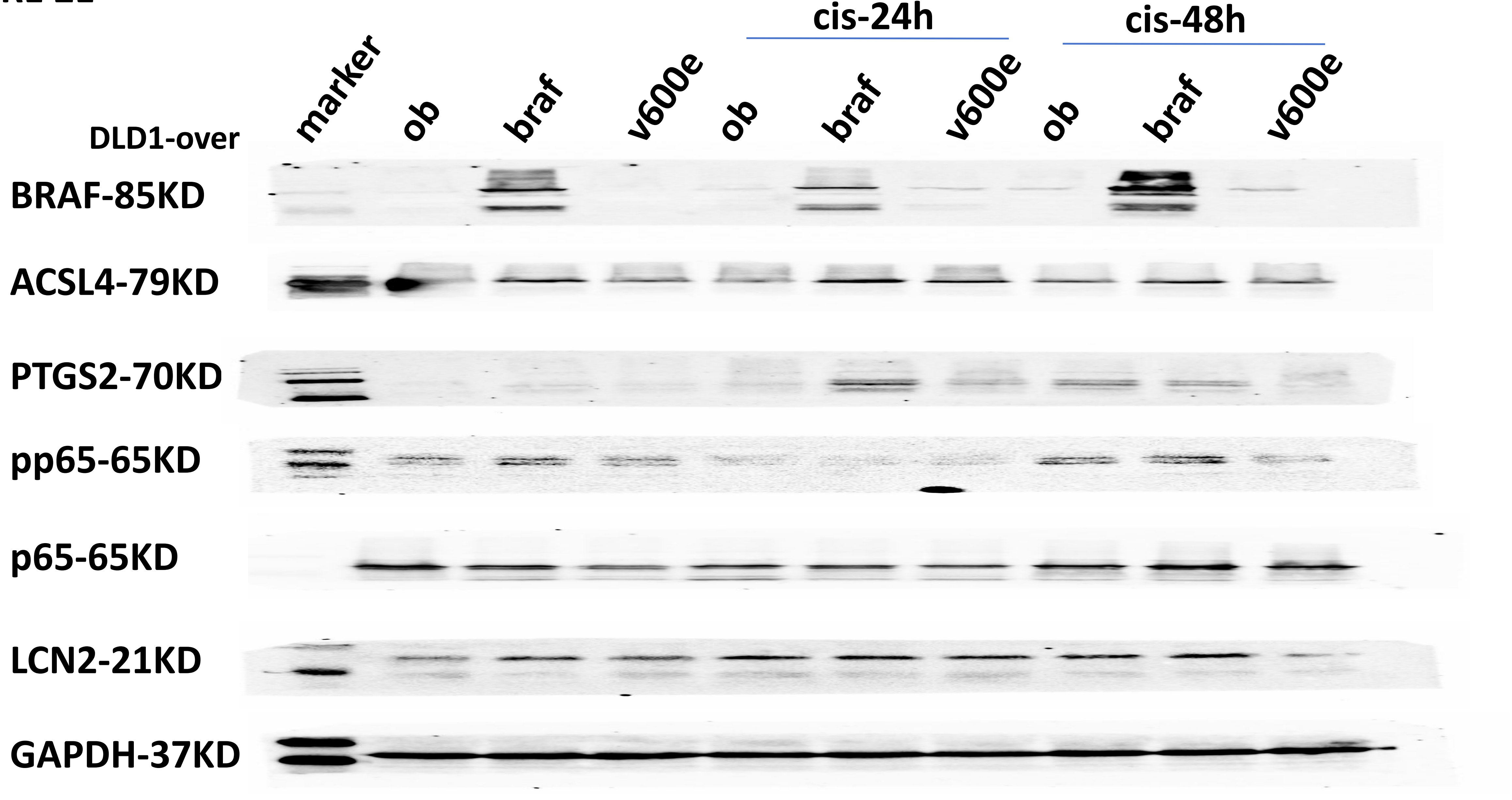
cis-24h
cis-48h
marker
ob
braf
v600e
ob
braf
v600e
ob
braf
v600e
DLD1-over
BRAF-85KD
ACSL4-79KD
PTGS2-70KD
pp65-65KD
p65-65KD
LCN2-21KD
GAPDH-37KD

FIGURE 2E

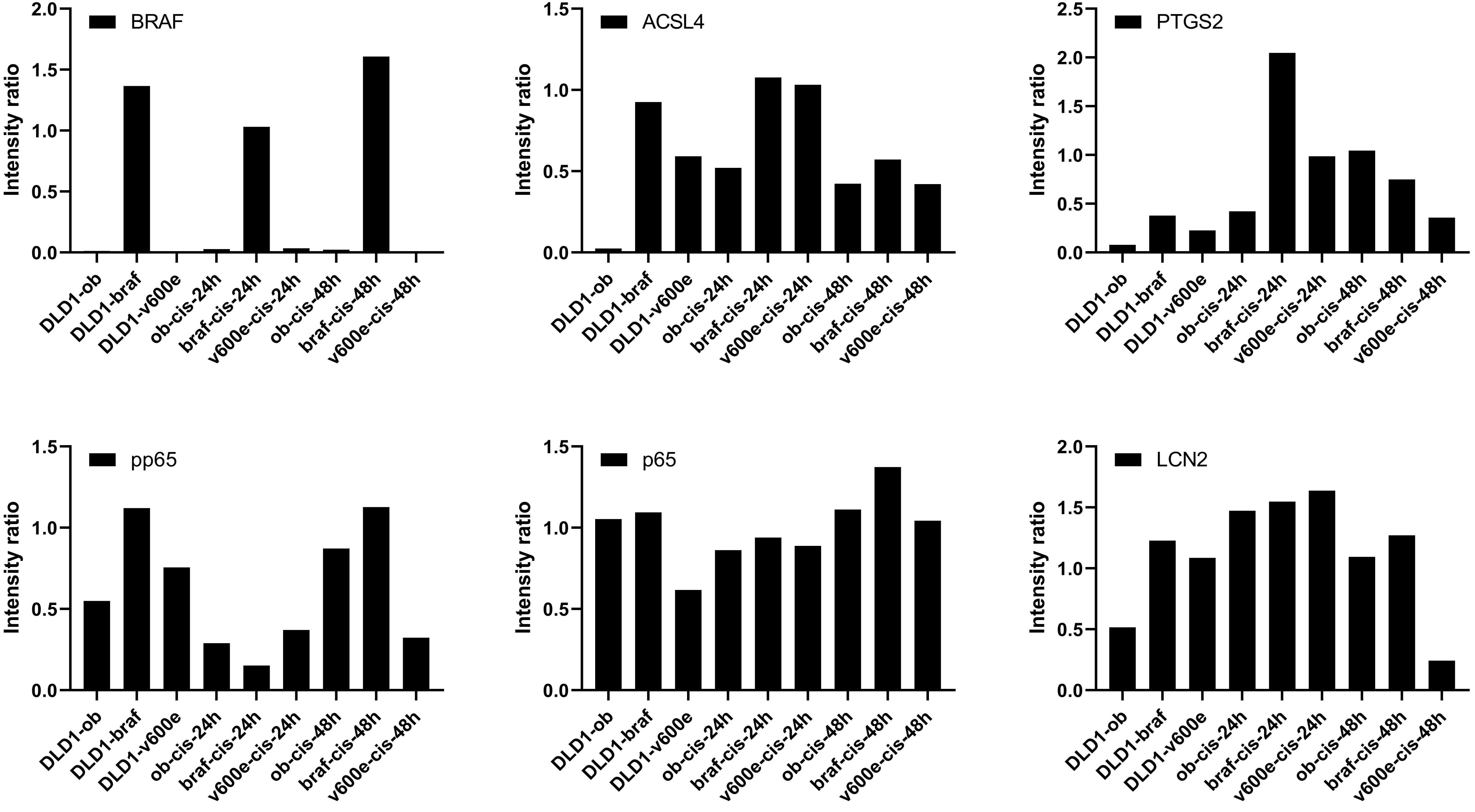
BRAF
ACSL4
PTGS2
pp65
p65
LCN2
Intensity ratio
0.0
0.5
1.0
1.5
2.0
2.5
DLD1-ob
DLD1-braf
DLD1-v600e
ob-cis-24h
braf-cis-24h
v600e-cis-24h
ob-cis-48h
braf-cis-48h
v600e-cis-48h

FIGURE 4B

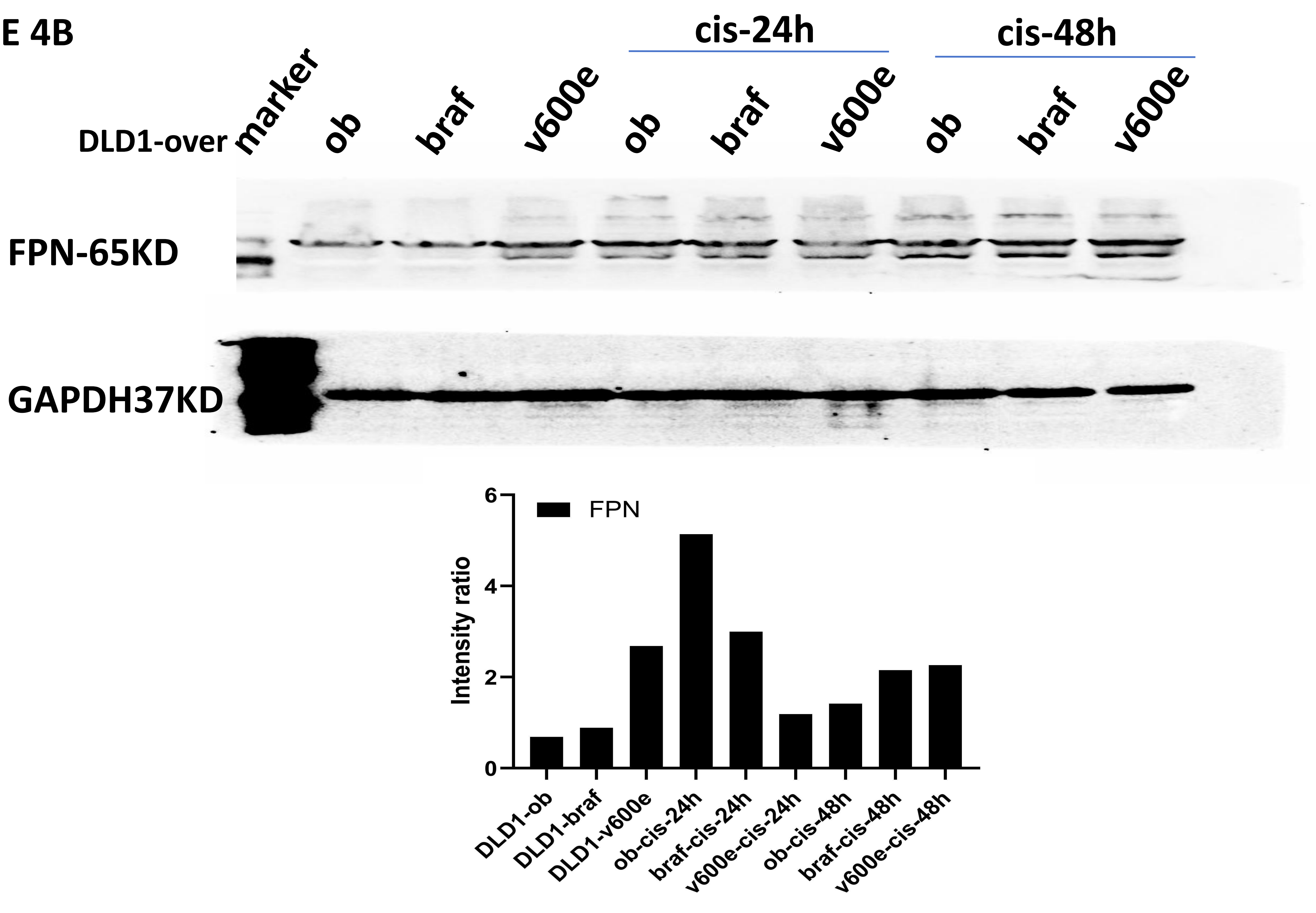
cis-24h
cis-48h
DLD1-over
marker
ob
braf
v600e
ob
braf
v600e
ob
braf
v600e
FPN-65KD
GAPDH37KD
FPN
Intensity ratio
6
4
2
0
DLD1-ob
DLD1-braf
DLD1-v600e
ob-cis-24h
braf-cis-24h
v600e-cis-24h
ob-cis-48h
braf-cis-48h
v600e-cis-48h

FIGURE 5A

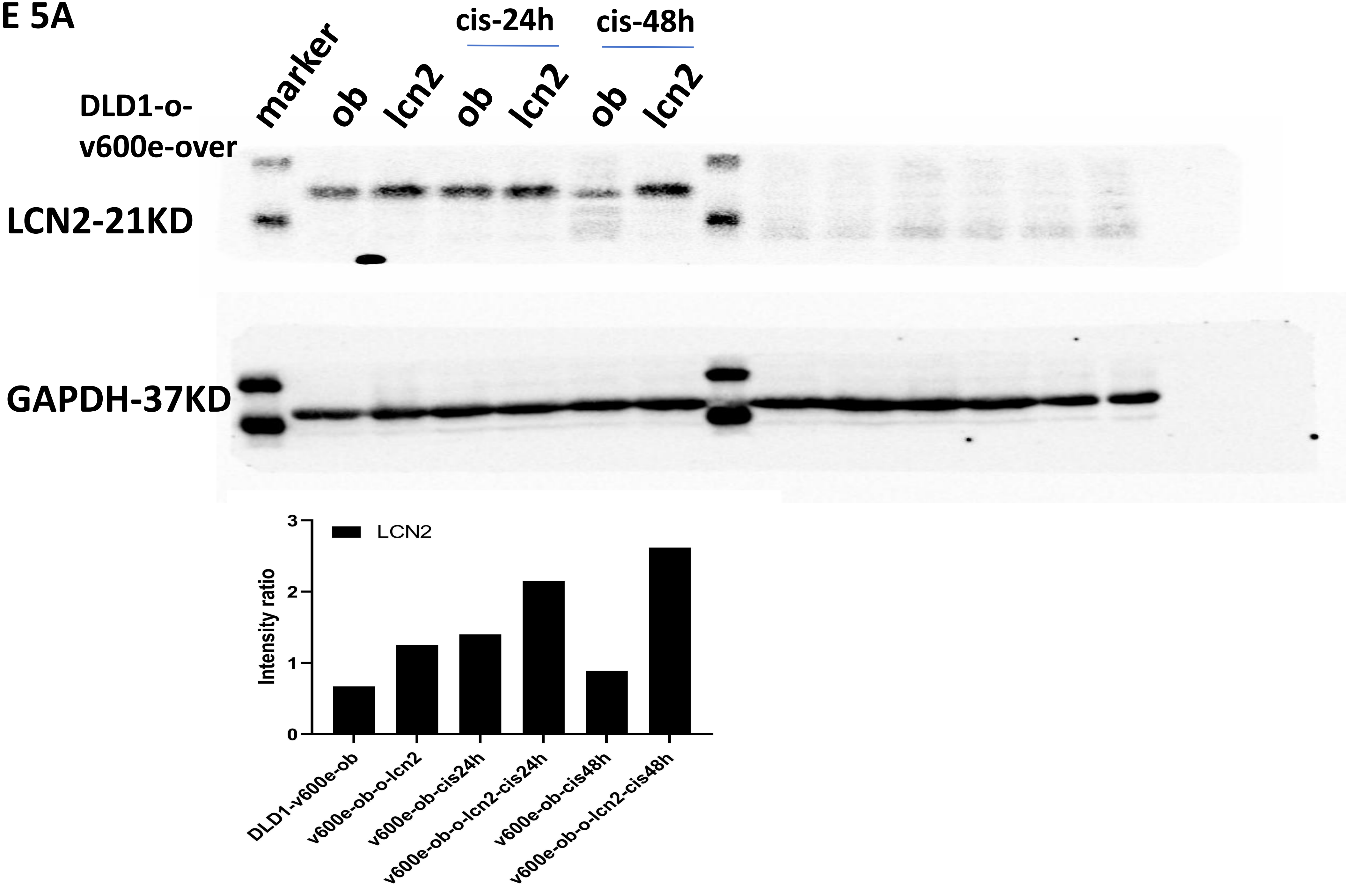
cis-24h
cis-48h
marker
ob
lcn2
ob
lcn2
ob
lcn2
DLD1-o-
v600e-over
LCN2-21KD
GAPDH-37KD
LCN2
Intensity ratio
3
2
1
0
DLD1-v600e-ob
v600e-ob-o-lcn2
v600e-ob-cis24h
v600e-ob-o-lcn2-cis24h
v600e-ob-cis48h
v600e-ob-o-lcn2-cis48h
